# Microbial Dynamics during the Removal of Linear Alkylbenzenesulfonate (LAS) under Anaerobic and Microaerobic Conditions with Different Co-substrates

**DOI:** 10.1021/acsomega.6c01030

**Published:** 2026-09-22

**Authors:** Maurício Guimarães de Oliveira, Vicente Elício Porfiro Sales Gonçalves da Silva, Amanda de Sousa e Silva, Fabricio Motteran, André Bezerra dos Santos

**Affiliations:** † Department of Hydraulic and Environmental Engineering, 28121 Federal University of Ceará, 60455-900 Fortaleza, Ceará, Brazil; ‡ Department of Civil Engineering, Centre for Technology and Geosciences, 28116 Federal University of Pernambuco, 50740-550 Recife, Brazil

## Abstract

Linear alkylbenzenesulfonate (LAS) is the most widely used anionic surfactant and is commonly found in wastewater due to its extensive use in detergents and cleaning products. Although LAS is biodegradable, its degradation in anaerobic environments remains a challenge, often requiring the addition of co-substrates and optimized operating conditions. This pioneering study evaluates the microbiological pathways and metabolisms underlying LAS biodegradation in UASB reactors operated under microaerated conditions, compared with strictly anaerobic systems. Furthermore, the effects of different co-substrates (ethanol and sucrose) on LAS removal efficiency and biogas production were also evaluated. The results indicate that microaeration, combined with sucrose as a co-substrate, significantly improves LAS removal, increasing efficiency by 34.3%. The study also revealed that microaeration influenced methane production, reducing its purity but increasing overall volumetric biogas production. Microaeration significantly influenced the microbial structure and function of the systems, depending on the co-substrate used. In the presence of ethanol, both systems showed a predominance of fermentative and methanogenic communities, with no significant differences in LAS removal. In contrast, with sucrose, microaeration promoted greater removal efficiency, associated with the selection of a specialized consortium composed of *Pleomorphomonas* and *Desulfovibrio*, capable of partially oxidizing the benzene ring and desulfonating the surfactant. This condition activated oxidative and sulfur metabolism pathways, while maintaining functional methanogenesis, resulting in a less diverse but metabolically more targeted and efficient community in LAS degradation. These findings highlight the potential of microaeration and co-substrate selection to optimize anaerobic treatment of LAS-containing wastewater while maintaining stable reactor performance.

## Graphical abstract

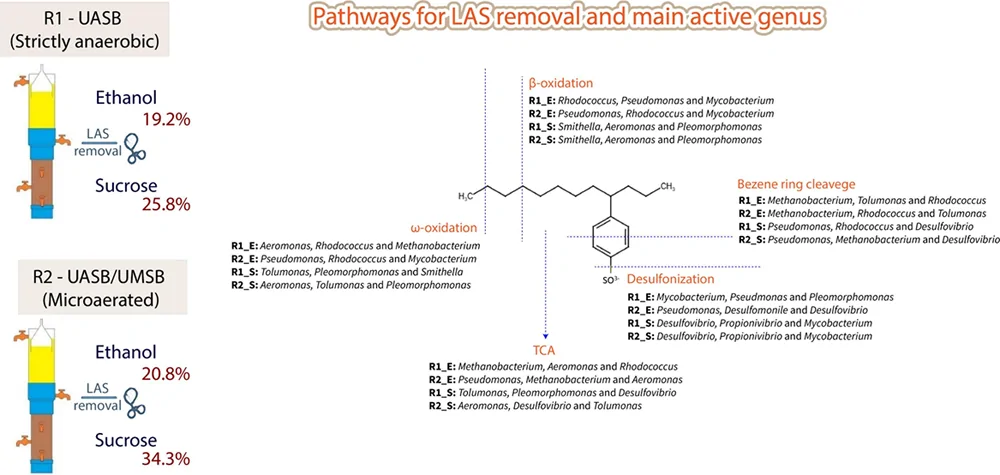

## 1 Introduction

Upflow Anaerobic Sludge Blanket (UASB) reactors are widely applied for domestic wastewater treatment and have increasingly been investigated for the removal of emerging contaminants (ECs), which are of concern due to their persistence and ecotoxicological effects.^1–3^ Among them, Linear Alkylbenzenesulfonate (LAS) is the most extensively used anionic surfactant worldwide and is frequently detected in domestic wastewater because of its widespread use in detergents, cleaning products, and personal care formulations.^4,5^ LAS concentrations in wastewater treatment plant influents typically range from 0.8 to 21 mg·L^–1^,^6^ and its occurrence in aquatic environments has been associated with foam formation, reduced oxygen transfer, and diminished light penetration.^4,7^

Although LAS can be removed under anaerobic conditions, with efficiencies in UASB reactors ranging between 30% and 91%, its biodegradation remains highly dependent on electron donor availability and the activity of specialized microbial consortia, which partly explains the large variability in removal efficiencies reported for UASB reactors.^5,7–9^ In this context, readily fermentable co-substrates such as ethanol and sucrose can enhance LAS removal by supplying reducing equivalents and metabolic intermediates that sustain syntrophic interactions and support co-metabolic degradation processes.^2,6^

The importance of these interactions stems from the fact that anaerobic LAS biodegradation is inherently more complex than its aerobic counterpart, as it relies on energetically less favorable pathways involving fumarate addition to the alkyl chain, followed by β-oxidation, aromatic ring cleavage, and desulfonation.^10^ Consequently, the complete transformation of LAS depends on the coordinated activity of multiple microbial populations, including members of the Synergistetes phylum as well as genera such as Pseudomonas and Desulfovibrio, which have frequently been associated with the degradation of aromatic and sulfonated intermediates in anaerobic environments.^6,11,12^ As a result, limitations in electron transfer, syntrophic cooperation, and the activation of key degradation steps often restrict LAS biodegradation under strictly anaerobic conditions.

Despite the growing number of studies investigating LAS degradation in anaerobic reactors, most efforts have focused on reactor configuration, hydraulic retention time, biomass adaptation, and the use of supplementary carbon sources. These studies have demonstrated that LAS removal can be achieved under anaerobic conditions, but the reported efficiencies remain highly variable and frequently limited by the slow degradation of aromatic intermediates, insufficient electron transfer, and the accumulation of partially transformed metabolites. Consequently, strategies capable of overcoming these biochemical bottlenecks while preserving the energetic advantages of anaerobic treatment remain an important research challenge.

Given that LAS degradation is typically more efficient under aerobic conditions, the strategic introduction of low-oxygen doses into anaerobic systems via microaeration emerges as a promising approach to enhance surfactant removal. Controlled microaerobic inputs can stimulate facultative and microaerotolerant microbial taxa, promote the partial oxidation of xenobiotic intermediates, and facilitate subsequent anaerobic transformations, all while preserving the redox conditions required for stable methanogenesis and biogas production.^13^

Microaeration has consistently enhanced the removal of diverse recalcitrant pollutants. For BTEX compounds, microaeration rates of 0.5–2.0 mL air·min^–1^ increased overall removal to above 83%, with benzene elimination rising by approximately 30% relative to anaerobic conditions.^14^ In the case of pharmaceuticals, applying microaeration in an Anaerobic Biofilm Blanket – Membrane Bioreactor system increased antibiotic degradation to 78% for ciprofloxacin and 88–91% for sulfamethoxazole, clearly surpassing the non-aerated reactor.^15^ For dyes, microaeration enabled the complete removal of aromatic amines from the dye DB22 in a UASB reactor, producing an effluent 16 times less toxic than that from the anaerobic control.^16^ Similarly, for hormones and pharmaceuticals, an airflow of 4 mL·min^–1^ increased removal to 90–94%, compared to less than 30% under strictly anaerobic operation.^17^ Microaeration is also widely applied for hydrogen sulfide oxidation in biogas treatment.^18^

Collectively, these studies indicate that microaeration acts as a process intensification strategy capable of selectively activating oxygen-dependent reactions that are inaccessible under strictly anaerobic conditions. By modifying local redox conditions, controlled oxygen supply can influence microbial community structure, extracellular polymeric substances production, and the distribution of metabolic functions responsible for contaminant transformation.^19–21^ Consequently, oxygen availability not only affects the overall extent of LAS removal, but also determines the dominant biodegradation routes involved in its mineralization. Recent studies have demonstrated that variations in oxygen supply can shift the balance between aerobic and anaerobic metabolic pathways, alter the abundance of key functional microorganisms, and modify the fate of LAS transformation intermediates.^22,23^ Together, these findings highlight that oxygen acts not merely as an electron acceptor, but as a key regulator of the biological processes governing LAS degradation. Nevertheless, despite these advances, the application of controlled oxygen supplementation for LAS removal in anaerobic reactors remains unexplored.

In addition, previous studies have shown that LAS degradation is strongly influenced by operational parameters that regulate microbial metabolism, including oxygen availability, nutrient balance, electron donor supply, and microbial community composition.^20,23,24^ Variations in these factors can substantially modify degradation pathways, extracellular polymeric substances accumulation, nitrogen transformation routes, and the abundance of key microbial populations involved in LAS mineralization.^20,24^ Although co-substrate supplementation and microaeration have independently been shown to enhance the biodegradation of recalcitrant compounds, their combined effects on anaerobic LAS removal remain unknown. This knowledge gap is particularly relevant because oxygen supplementation and co-substrate availability simultaneously affect electron transfer, syntrophic interactions, oxidative activation reactions, and microbial adaptation to surfactant stress, all of which are key processes governing LAS transformation.

Therefore, this study evaluated the combined influence of controlled microaeration and readily degradable co-substrates on LAS removal in UASB reactors treating synthetic domestic wastewater. Particular attention was given to understanding whether oxygen supplementation could overcome metabolic bottlenecks traditionally associated with anaerobic LAS degradation and whether the nature of the co-substrate influences this response. In addition to reactor performance, the metabolic pathways potentially involved in LAS transformation were investigated to provide a mechanistic interpretation of the observed behavior.

## 2 Materials and Methods

The present study was structured into two distinct experiments. The first experiment involved the continuous operation of two reactors to evaluate the effects of microaeration and the use of different co-substrates (ethanol and sucrose) on LAS removal. The second experiment consisted of a batch assay designed to preliminarily assess the role of primary fermentative microorganisms (represented by sucrose), secondary fermentative microorganisms (represented by ethanol), and acetoclastic methanogenic archaea (represented by acetate), as well as abiotic and endogenous processes, in relation to LAS removal efficiencies in continuous-flow experiments under the tested operational conditions.

### 2.1 Continuous Operation Experiment Setup

Two cylindrical acrylic UASB reactors (Figure S1) were operated in parallel at the laboratory scale, with a working volume of 3.25 L. Both reactors were inoculated with 1.6 L of sludge and operated under the same conditions, differing only in the microaeration insert: R1, traditional UASB, R2, microaerated UASB (renamed to UMSB – Upflow Microaerobic Sludge Blanket).

The inoculum was sludge from a UASB reactor treating sewage in Caucaia, Brazil. Total solids, total volatile solids and total fixed solids in the inoculum sludge were 48 ± 3, 25 ± 1, and 23 ± 1 g·L^–1^, respectively.

Microaeration in R2 was performed with synthetic air (80% N_2_:20% O_2_, White Martins, Brazil) at its base using a mass flow controller (GFC17, Cole-Parmer, USA). The microaeration air dose used in R2 was approximately 0.7 L_air_·min^–1^, corresponding to 0.1 LO_2_. Lfeed^–1^.d^–1^, which was kept constant during the experimental period. The microaeration dose was selected based on the best results reported by Nascimento et al. (2021), who evaluated the removal of emerging sulfonated components. The reactors were fed by two peristaltic pumps (ColeParmer MasterFlex L/S 7522-30, USA) at a flow rate of 6.77 mL·min^–1^, resulting in an HRT of 8 h, a value similar to that used in sewage treatment plants.^25^

The experiment was conducted in two sequential phases, each lasting 45 days, with a 45-day stabilization interval in between. In the first phase, ethanol was used as the co-substrate, and in the second phase, sucrose was introduced to evaluate its impact on system performance. During the stabilization interval, the reactors were fed with ethanol in the absence of LAS to reset microbial activity and ensure a clear transition between phases. This structured approach allowed for a systematic comparison of the effects of different co-substrates on organic matter degradation and LAS removal under both anaerobic and microaerobic conditions.

### 2.2 Batch Experiment

At the end of the first operational phase, sludge samples from both reactors were collected, homogenized, and used in the presence of acetate, ethanol, and sucrose to assess LAS degradation and methanogenesis. The experiment was conducted in triplicate, following the methodology described by Angelidaki et al.^26^ Anaerobic reactors consisted of 300 mL borosilicate glass flasks with a working volume of 200 mL and a 100 mL headspace, maintained under continuous agitation at 150 rpm and 35 ± 1 °C.

A substrate-to-microorganism ratio (S/M) of 0.5 gCOD·gVS^–1^ was adopted, based on a final concentration of 1 gCOD·L^–1^ for the substrate and 2.0 gVS·L^–1^ for the inoculated sludge. The basal medium included LAS (15 mg·L^–1^), macro- and micronutrients, and sodium bicarbonate (1 g·L^–1^) as a buffering agent. Two control groups were established: (i) endogenous control (containing LAS, sludge, and basal medium, but no additional carbon source), and (ii) chemical control (containing only LAS and distilled water), as illustrated in Figure S2.

All reactors were sealed with butyl rubber stoppers and purged with nitrogen (N_2_) for approximately 1 min to ensure anaerobic conditions. The experiment was conducted over 174 h, a period determined based on the cessation of CH_4_ production, indicating the completion of the anaerobic digestion process in batch mode.

### 2.3 Synthetic Wastewater

The synthetic wastewater consisted of an aqueous solution containing LAS (15 mg·L^–1^), ethanol (1 gCOD.L^–1^) (Phase I), sucrose (1 gCOD.L^–1^) (Phase II), basal medium (macro and micronutrients) prepared according to Firmino et al.^27^ and sodium bicarbonate (1 g·L^–1^) to maintain the pH close to 7.0. All reagents were used as purchased without further purification. The synthetic influent was kept refrigerated at 4 °C to prevent natural biodegradation of organic matter.

### 2.4 Chemical Analysis

Chemical oxygen demand (COD), pH, total suspended solids, and total solids content were determined according to APHA.^28^ The quantification of CH_4_, CO_2_, H_2_, and H_2_S in the biogas was determined by gas chromatography with ionization detection by dielectric barrier discharge (GC BID-2010 Plus, Shimadzu Corporation, Japan), equipped with GS-GASPRO column (60 m × 0.32 mm) (Agilent Technologies Inc., USA). Helium gas was used as the carrier gas (White Martins LTDA, Brazil) at a flow rate of 2 mL·min^–1^, with a run time of 9 min. Oven, injector, and detector temperatures were 50, 100, and 250 °C, respectively. O_2_ and N_2_ were quantified by gas chromatography with thermal conductivity detection (GC-TCD) (GC-17A, Shimadzu Corporation, Japan). The biogas sample (1.0 mL) was injected in splitless mode, and chromatographic separation was performed on a Mol Sieve 5A PLOT column (30 m, 0.32 mm ID) (Restek Corporation, USA). Oven, injector, and detector temperatures were 35, 40, and 230 °C, respectively. Helium (White Martins LTDA, Brazil) was used as the carrier gas at a flow rate of 7 mL·min^–1^, and the run time was 5 min.

LAS concentrations were determined according to the methodology adapted from Silva et al.^29^ by high-performance liquid chromatography (HPLC) using a Shimadzu system (CBM-20A, SPD-20A, CTO-20A, SIL-20A, and LC-20AT) equipped with a reversed-phase C18 column from Shimadzu. The mobile phase was water with 5 mmol·L^–1^ ammonium acetate (solvent A) and acetonitrile (solvent B); the mobile phase flow rate was 1 mL·min^–1^, the sample volume was 50 μL, the temperature of the thermostated column compartment was 40 °C, and UV-Vis detection at 230 nm. A gradient was applied to the mobile phase to separate the LAS constituents. It started with 45% solvent B, and its concentration began to rise after 5 min, reaching 60% in 20 min. The concentration then returned to 45% after 1 min and remained constant for 6 min.

### 2.5 Microbiological Analysis

At the beginning of the experiment and the end of each phase, sludge samples from both reactors were collected to analyze the microbial changes. For this purpose, sludge samples were collected from different reactors’ heights, characterizing a biomass composite sample. 0.25 g of fresh sample (total wet weight) was used to extract DNA from microorganisms using the PowerSoil® DNA Isolation Kit (QIAGEN, Hilden, Alemanha), following the manufacturer’s instructions. Based on the V4 region of the 16S rRNA gene, amplicon sequencing was performed in triplicate according to the Illumina protocol, using specific primers (515*F*/806R).^30,31^

After indexing, the PCR products (amplicons) were purified using Agencourt AMPure XP-PCR purification beads (Beckman Coulter, Fisher Scientific, USA), according to the product manual, and quantified using the dsDNA BR assay kit (Invitrogen, ThermoFisher Scientific, USA) on a Qubit 2.0 fluorimeter (Invitrogen, ThermoFisher Scientific, USA). The amplicons were sequenced using the MiSeq 300-cycle chemistry reagent kit (Illumina, USA) on the MiSeq desktop sequencer (Illumina, USA) at the Genomics and Bioinformatics Center (CeGenBio) at UFC.

The sequencing data were analyzed using bioinformatics tools. The DADA2 package v1.28.0 ^32^ was used to filter and assemble reads into amplicon sequence variants (ASVs). The obtained sequences were taxonomically classified using version 5.1 of the MiDAS database, which catalogs microorganisms identified in anaerobic digesters, with the assign Taxonomy method of the DADA2 package. Alpha diversity indices were estimated with the estimate_richness method of the Phyloseq package v1.44.0. The raw sequence data obtained in this study have been deposited in the National Center for Biotechnology Information (NCBI) BioProject database, ID PRJNA836880.

To infer the metabolic capabilities of the microbial communities present in the reactors samples, the ASV-based 16S rRNA abundance data were analyzed using PICRUSt2 (Phylogenetic Investigation of Communities by Reconstruction of Unobserved States). Functional predictions were generated based on KEGG (Kyoto Encyclopedia of Genes and Genomes) pathways, using EC (Enzyme Commission) numbers and KO (KEGG Orthology) identifiers to assign putative gene functions.^33–36^

### 2.6 Statistical Methods

The Mann–Whitney nonparametric test, which do not require specific distributional assumptions, were used to compare reactor performance across the different experimental periods at the 5% significance level (*p* < 0.05).

## 3 Results and Discussion

### 3.1 Linear Alkylbenzenesulfonate (LAS) Removal

To evaluate the effects of co-substrate type and microaeration on reactor performance, LAS removal was monitored during both the acclimatization and stability phases (Figure 1). During acclimatization, all reactors exhibited considerable variability, with standard deviations exceeding 10% of the mean removal efficiency, indicating that the microbial communities were still adapting to the operational conditions. In contrast, LAS removal became more stable after reactor stabilization, particularly in the sucrose-fed systems, reflecting the establishment of a more robust microbial consortium.

**Figure 1:**
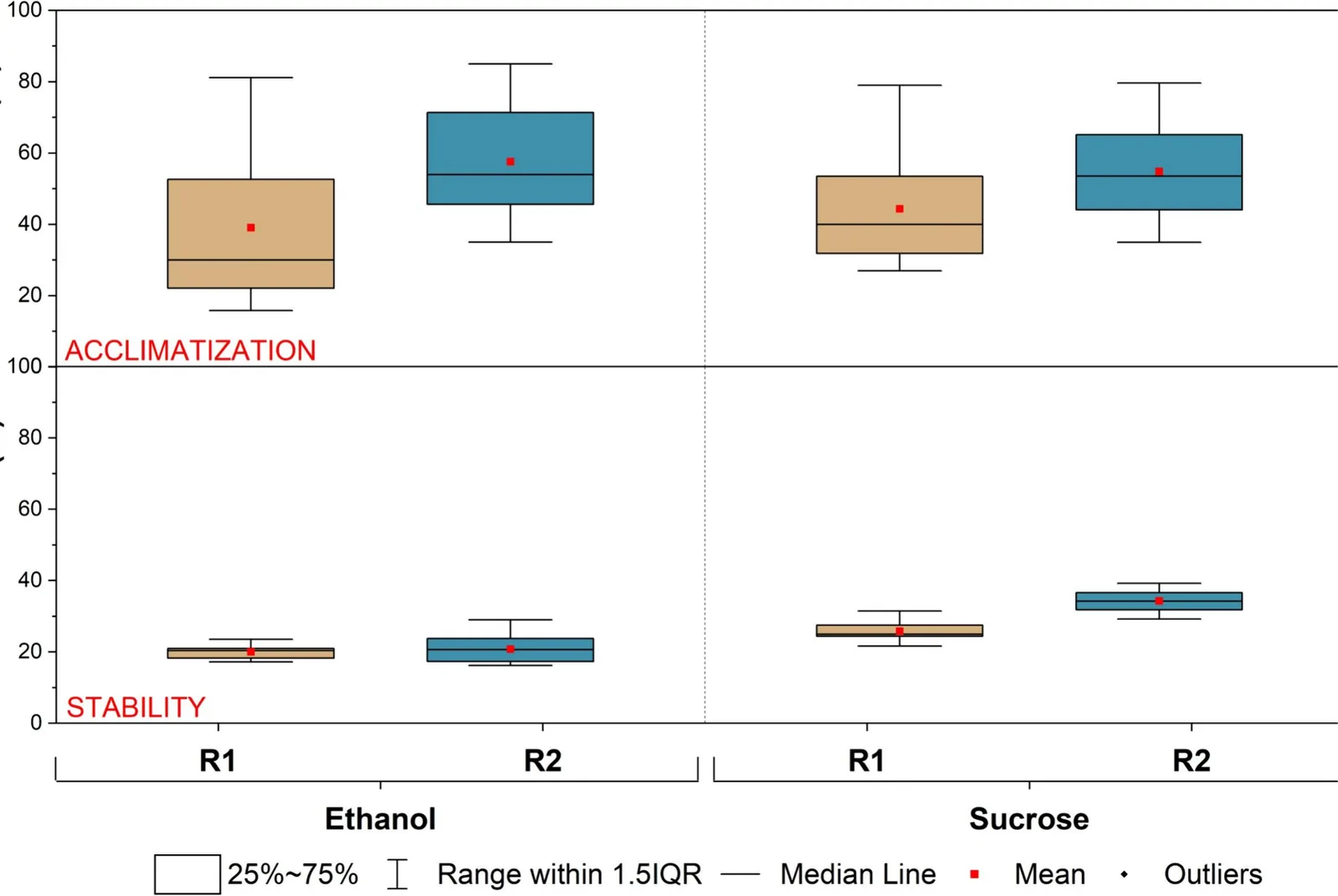
Removal of LAS in anaerobic (R1) and microaerobic (R2) reactors throughout the acclimatization and stability period.

During the stability period under ethanol feeding, LAS removal averaged 19.2 ± 3.2% in the control reactor (R1) and 20.8 ± 4.1% in the microaerated reactor (R2), with no statistically significant difference between reactors (p = 0.32). When sucrose replaced ethanol as the co-substrate, LAS removal increased significantly in both systems. In R1, removal efficiency increased from 19.2 to 25.8% (p = 0.034), whereas in R2 it increased from 20.8 to 34.3% (p < 0.001). The highest removal efficiency was therefore achieved by the combination of microaeration and sucrose supplementation, suggesting a synergistic effect between oxygen availability and co-substrate composition.

These findings indicate that co-substrate selection exerted a greater influence on LAS degradation than microaeration alone. Sucrose, as a readily fermentable carbohydrate, likely promoted faster production of volatile fatty acids and supported a broader range of fermentative, facultative, and syntrophic microorganisms, creating metabolic conditions more favorable for surfactant biodegradation than those established with ethanol.^37,38^ The effect was particularly evident under microaerated conditions, where the enhanced metabolic flexibility may have facilitated the coexistence of oxidative and anaerobic pathways potentially involved in LAS degradation.

The LAS removal efficiencies obtained under ethanol feeding were higher than those reported by Okada et al.,^8^ who achieved 9.0 ± 2.4% removal in UASB reactors operated under comparable LAS concentrations using a co-substrate mixture of ethanol, methanol, and yeast extract. Moreover, although the same authors observed that increasing co-substrate complexity enhanced LAS removal, reaching up to 50% with yeast extract supplementation, the present study demonstrates that sucrose alone was sufficient to significantly improve LAS degradation under shorter hydraulic retention time conditions (8 h). These observations reinforce the importance of co-substrate characteristics in determining the metabolic potential of anaerobic communities involved in LAS degradation.

In contrast, Macedo et al.^39^ reported higher LAS removal with ethanol than with a sucrose–ethanol mixture in a fluidized-bed reactor. This discrepancy is likely related to differences in reactor configuration and operating conditions, highlighting that the effect of co-substrate selection depends on the operational environment.

From a practical perspective, the superior performance achieved by the microaerated reactor supplemented with sucrose demonstrates the potential of combining low-intensity oxygen supply with an appropriate co-substrate as a process intensification strategy for UASB reactors treating LAS-containing domestic wastewater. Because the improvement was obtained under an HRT of 8 h, a hydraulic condition compatible with full-scale domestic wastewater treatment plants, this approach may enhance LAS removal without requiring larger reactor volumes or major modifications to conventional UASB operation.

### 3.2 Volumetric Biogas Production

To investigate the effects of co-substrate type and microaeration on methane generation, volumetric biogas production and methane content were evaluated. Figure 2 shows volumetric biogas and methane production in reactors operated with sucrose and ethanol as substrates. Monitoring data show that both reactors achieved higher volumetric methane production with sucrose. Under ethanol feeding, R1 (conventional UASB reactor) produced an average of 1.8 ± 0.4 L·day^–1^, whereas the microaerated reactor (R2) reached 2.7 ± 0.5 L·day^–1^. With sucrose, both reactors showed similar performance: R1 yielded 2.7 ± 0.3 L·day^–1^, and R2 achieved 2.7 ± 0.4 L·day^–1^. These findings indicate that sucrose, as a more complex substrate, supported more uniform methane production across configurations.

**Figure 2:**
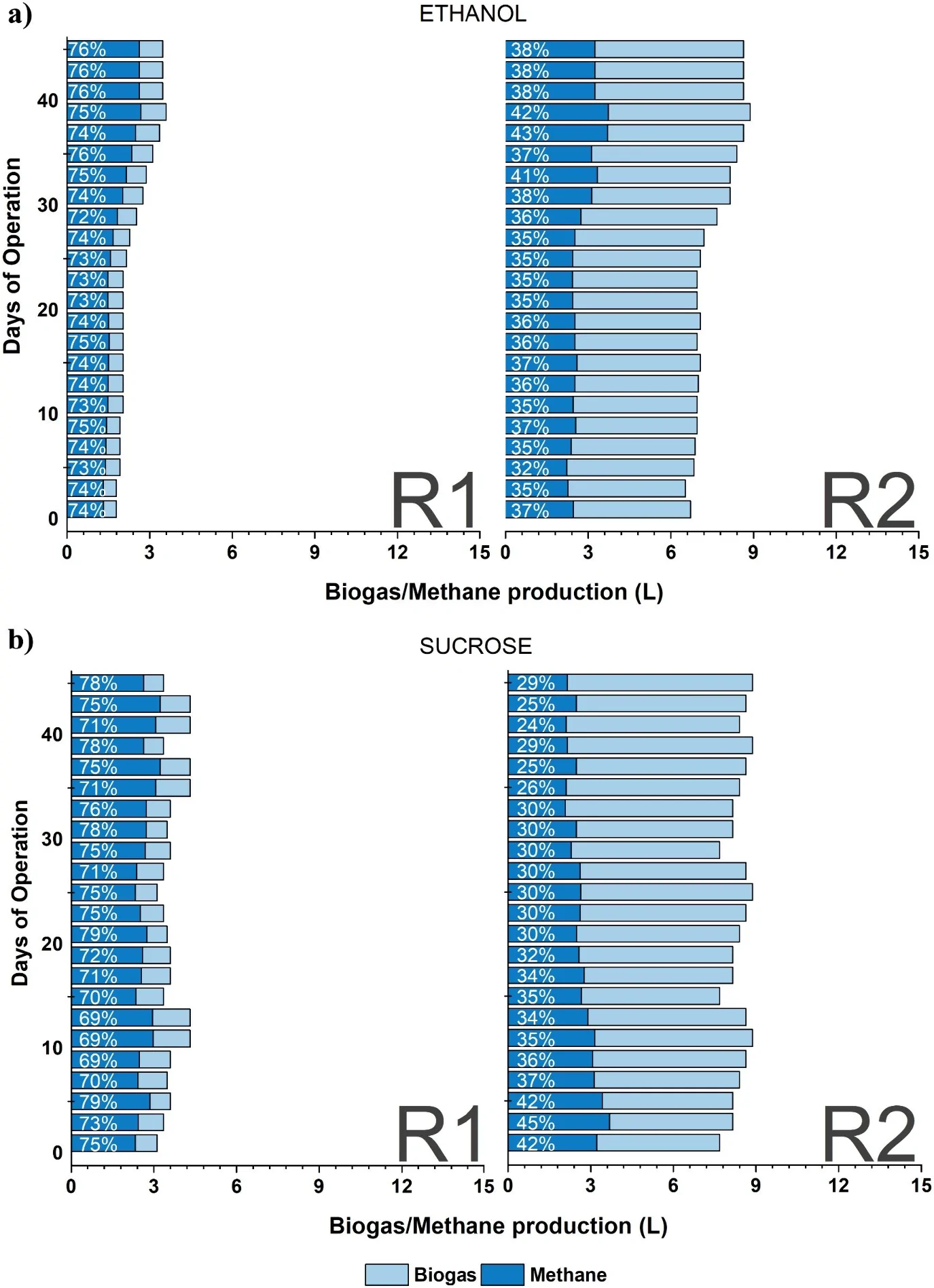
Biogas and methane production from reactors throughout the Ethanol (a) and Sucrose (b) phases.

Sucrose, a disaccharide composed of glucose and fructose, is hydrolyzed by hydrolytic bacteria into readily fermentable monosaccharides. These are processed through glycolysis into organic acids, which are subsequently transformed into acetate and methane by acetoclastic methanogens.^38^ Additionally, monosaccharides are converted into hydrogen (H_2_) and carbon dioxide (CO_2_), which hydrogenotrophic methanogens further reduce to methane. Ethanol, in contrast, undergoes a narrower metabolic route, being directly oxidized into acetate or H_2_ + CO_2_ by acetogenic bacteria prior to methanogenesis.^40^

Average methane concentration in biogas produced by R1 reached 73.9% during ethanol and 73.4% during sucrose operation, values exceeding the typical range for conventional anaerobic digestion (50–70%),^5^ reflecting the absence of oxygen and the dominance of methanogenic activity.

In contrast, microaerated systems showed lower methane content, averaging 36.6% (ethanol) and 32.7% (sucrose). For biogas to be considered a suitable energy source, the methane concentration must exceed 45%.^41^ This reduction is primarily due to dilution from air injection, along with possible shifts in microbial community structure induced by oxygen.

These trends were consistent with findings by Nascimento et al.^17^ and Oliveira et al.,^42^ who reported that microaeration enhances organic matter removal but compromises methane purity. Because the lower methane concentration mainly resulted from biogas dilution rather than inhibition of methanogenic activity, strategies such as controlled oxygen dosing, intermittent microaeration, or oxygen-enriched streams may reduce this effect while preserving the oxidative microzones required for LAS transformation and the energetic advantage of anaerobic treatment.

### 3.3 Batch Experiment

A batch experiment was conducted under anaerobic conditions to preliminarily assess the role of primary fermentative microorganisms (represented by sucrose), secondary fermentative microorganisms (represented by ethanol), and acetoclastic methanogenic archaea (represented by acetate), as well as abiotic and endogenous processes, in relation to LAS removal efficiencies in continuous-flow experiments under the tested redox conditions.

Endogenous controls revealed that 20% of LAS was adsorbed onto the biomass under all tested conditions, whereas the chemical control showed no abiotic LAS removal. Therefore, LAS removal was attributed to biological processes, whose efficiency depended on the type of co-substrate present.

LAS removal efficiencies from the liquid phase after 174 h of reactor operation were 80.7 ± 4.2%, 57.6 ± 2.9%, 52.2 ± 2.5%, and 59.1 ± 3.2% for the sucrose, ethanol, acetate, and endogenous series, respectively (Figure S3). In contrast, no LAS removal was observed in the chemical control series, which lacked both co-substrate and biomass, indicating that LAS is not self-biodegradable under the conditions tested.

LAS removal rates were 0.26 ± 0.4 mg·L^–1^·h^–1^ (sucrose), 0.16 ± 0.2 mg·L^–1^·h^–1^ (ethanol), 0.18 ± 0.3 mg·L^–1^·h^–1^ (acetate), and 0.16 ± 0.2 mg·L^–1^·h^–1^ (endogenous). These results suggest that co-substrates influence the assimilation rate of organic matter, with sucrose supporting higher LAS degradation rates than the other conditions, including the endogenous control.

Among the tested co-substrates, the acetate series exhibited the lowest LAS removal efficiency. This may be attributed to the metabolic limitations or sensitivity of microorganisms, particularly acetoclastic methanogens, to the anionic surfactant.^6^ Granatto et al.^43^ also noted that acetoclastic methanogens are more susceptible to surfactant toxicity, whereas hydrogenotrophic archaea are generally more resilient.

An important point to note was the high LAS removal observed in the endogenous control, indicating that in the absence of more efficient substrates for co-metabolic processes, such as sucrose or ethanol, LAS may be directly utilized by the microorganisms present or degraded through alternative co-metabolic pathways involving exopolysaccharides or substrates derived from endogenous metabolism.

### 3.4 Molecular Biology

#### 3.4.1 Microbial Community Richness and Diversity

To evaluate how co-substrate type and microaeration affected microbial richness and diversity, alpha-diversity indices and ASV distribution were analyzed. In general, although richness and diversity indices were higher in the Bacteria domain, they showed distinct behaviors within the Archaea (Figure 3a) and Bacteria (Figure 3b) domains, modulated by the nature of the co-substrate and the presence of residual oxygen introduced in R2.

**Figure 3:**
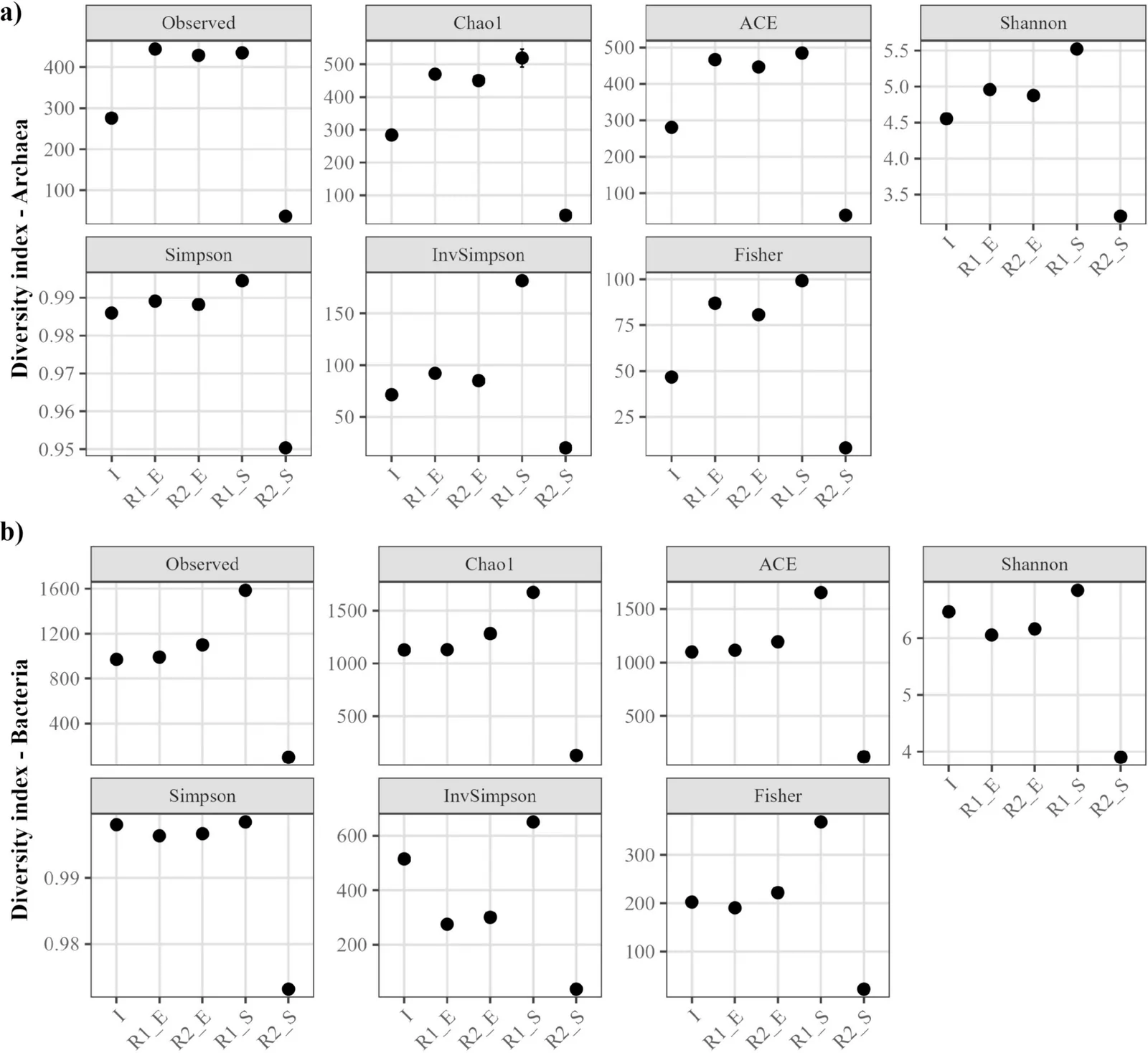
Diversity and richness indices separated by domain: a) Archaea and b) Bacteria.

For the Archaea domain, a consistent pattern was observed across all co-substrates. The conventional reactor (R1) showed greater richness and diversity, suggesting a more complex and balanced methanogenic community under strictly anaerobic conditions. The greater diversity of archaea in R1 may be associated with greater functional redundancy and trophic stability typical of consolidated anaerobic systems, but did not necessarily reflect greater efficiency in LAS degradation. This is because methanogenic archaea, although essential in the final stage of anaerobic digestion, predominantly convert simple intermediates into methane and do not directly participate in the oxidative reactions involved in LAS breakdown.^8,43^

In the bacterial domain, however, the response was more sensitive to the type of co-substrate. When ethanol was used, the microaerated reactor (R2) showed greater bacterial richness and diversity, indicating that the controlled oxygen availability favored the growth of facultative and microaerophilic groups that could coexist with strict anaerobes. This increased diversity may have broadened the system’s metabolic spectrum, although, in this case, LAS removal did not show a statistically significant difference between the reactors. This result suggests that, under ethanol, LAS degradation occurred mainly through anaerobic mechanisms, dependent on specific consortia already well adapted to the surfactant, and that the presence of residual oxygen did not represent a significant metabolic advantage. Furthermore, ethanol, being an easily assimilated substrate, tends to direct metabolism towards rapid fermentative and acetogenic pathways, reducing the residence time of aromatic intermediates derived from LAS and, therefore, limiting the potential benefit of microaeration in the partial oxidation of these compounds.^39,44^

In the presence of sucrose, a pronounced shift was observed in both the microbial community structure and system performance. In the microaerated reactor, richness and diversity indices decreased markedly for both Archaea and Bacteria, indicating the emergence of a more specialized community. This pattern is consistent with the fact that sucrose (hydrolyzed into glucose and fructose) is rapidly assimilated by bacteria equipped with highly efficient metabolic machinery for sugar utilization. As these sugars provide a high energetic yield and enable rapid ATP and biomass production, microorganisms with higher growth rates tend to outcompete slower-growing taxa, leading to reductions in diversity and richness and, in some cases, increased dominance within the community. Importantly, this community streamlining coincided with a substantial increase in LAS removal, particularly in R2, suggesting that the selective advantage conferred by sucrose assimilation favored microbial groups potentially associated with surfactant degradation.

This inverse relationship between diversity and performance suggests a process of functional selection, in which microaeration, associated with a more complex substrate such as sucrose, favored the enrichment of populations potentially associated with aromatic compound degradation. Limited oxygenation may have stimulated the activity of facultative bacteria involved in the initial steps of benzene ring oxidation, promoting the conversion of recalcitrant intermediates into molecules more assimilable by subsequent anaerobes, as already observed by other authors.^45,46^ Thus, the reduction in diversity reflected a more targeted and functionally efficient community, adapted to the degradation of LAS under controlled microaeration conditions.

In the microaerated reactor, this gradient may have intensified, creating complementary niches between microaerobic and anaerobic zones, which favor syntrophic pathways between partial oxidants and residual sulfate or nitrate reducers, as well as oxygen-tolerant methanogens. Thus, the greater removal of LAS observed in R2 with sucrose results not only from the presence of oxygen, but also from the interaction between the type of co-substrate and the consequent restructuring of the microbial community towards a more specialized and synergistic consortium.

The Venn diagram analysis corroborated the diversity patterns observed in the richness and diversity indices (Figure 4). R1 operated with sucrose exhibited the highest number of unique ASVs (1762), indicating a more heterogeneous microbial community under strictly anaerobic conditions. In contrast, R2 with sucrose presented only 91 unique ASVs and no ASVs shared with the inoculum, reflecting a strong microbial selection driven by microaeration and complete community restructuring. This specialized community coincided with the highest LAS removal observed in the study. Moreover, the presence of only 16 ASVs shared between the inoculum and all reactors indicates that both co-substrate type and microaeration acted as major selective forces shaping the microbial community. Overall, ethanol maintained more similar communities, whereas sucrose under microaeration promoted a less diverse but functionally more specialized consortium.

**Figure 4:**
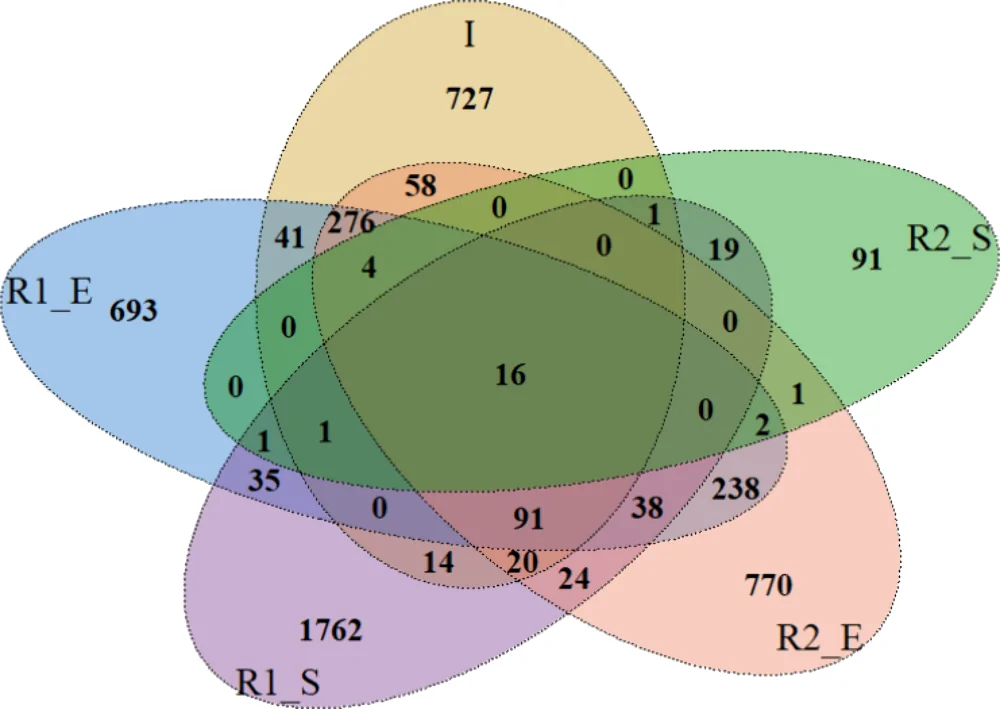
Venn diagram for each system operated, including the starting inoculum.

#### 3.4.2 Microbial Community Structure

To determine how co-substrate type and microaeration shaped the microbial community, the taxonomic composition of each reactor was analyzed (Figure 5).

**Figure 5:**
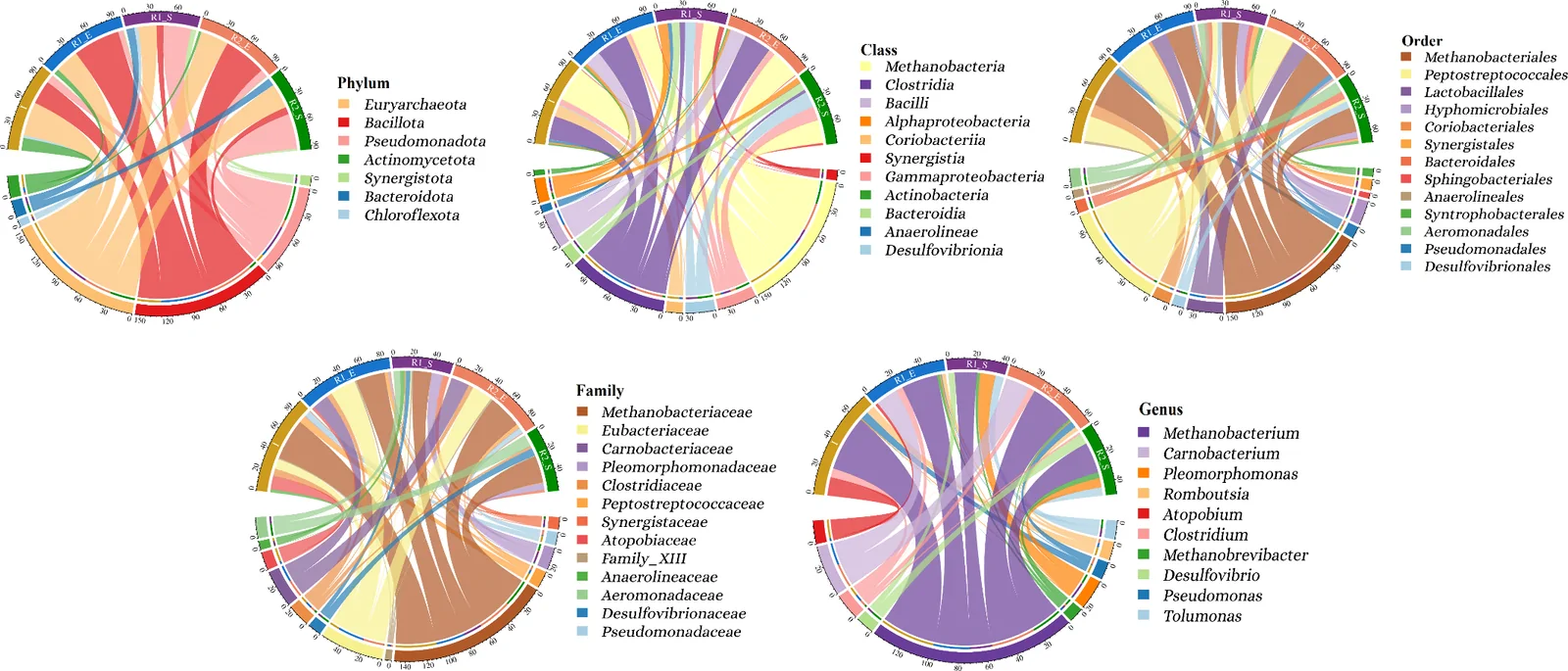
Structure of the microbial community in terms of phylum, class, order, family, and genus.

In systems with ethanol, the phylum *Bacillota* was the most dominant, especially in R1, and was mainly represented by the classes *Clostridia* and *Bacilli* and the families *Eubacteriaceae* and *Carnobacteriaceae*. In the anaerobic LAS removal experiments by Costa et al.,^9^ a predominance of the phylum *Bacillota* was also observed. Delforno et al.^47^ also found a greater abundance of *Bacillota* in the biomass of the bed of an expanded granular bed sludge reactor treating effluent with 14 mg·L^–1^ of LAS, ethanol, and methanol.

The predominance of the *Clostridium* and *Carnobacterium* genera in these reactors indicates the prevalence of typical fermentative pathways focused on the conversion of simple substrates into volatile fatty acids, H_2_, and CO_2_. These groups play a significant role in the acidogenic stage by oxidizing organic acids such as acetate, butyrate, and propionate, but are inefficient at degrading recalcitrant molecules such as LAS.^38,48^

The slight reduction of *Clostridium* with microaeration suggests that residual oxygen exerted a selective pressure against strict anaerobes, without, however, introducing groups capable of degrading LAS more effectively. This explains the absence of a statistical difference in LAS removal between R1 and R2 with ethanol, as the metabolism was dominated by redundant fermentative pathways, with a low contribution from initial oxidative pathways.

Upon replacing the co-substrate with sucrose, a significant microbiological transition occurred. The phylum *Pseudomonadota* became dominant, mainly in the microaerated R2, accompanied by an increase in the *Alpha*-, *Gamma*-, and *Desulfovibrionia* classes. This change is directly associated with the increase in LAS removal, as these groups harbor genera with documented capacity to oxidize aromatic compounds and surfactants. *Pseudomonadota* has been the most abundant phylum commonly found in anaerobic systems treating effluents with surfactants, especially with sucrose as a co-substrate.^9,12,38^

In R2_S, the increase in the genera *Pleomorphomonas* (family *Pleomorphomonadaceae*, *Alphaproteobacteria*) and *Desulfovibrio* (family *Desulfovibrionaceae*, *Desulfovibrionia*) evidences the formation of a specialized microaerophilic-syntrophic consortium. *Pleomorphomonas* includes species capable of metabolizing aromatic compounds under microaerobic conditions. At the same time, *Desulfovibrio* is known to act in sulfate reduction processes and degradation of sulfonated intermediates, being considered one of the most abundant genera associated with LAS degradation.^12,38,49^ The co-occurrence of these genera under microaeration suggests a potential sequential transformation of LAS, in which *Pleomorphomonas* may contribute to the initial oxidation of aromatic structures, whereas *Desulfovibrio* may participate in subsequent sulfonate transformation. This synergy explains the superior performance of R2_S in removing LAS, compared with R1_S, where the absence of oxygen limited the formation of more biodegradable intermediates.

Furthermore, the stimulation of Pseudomonadaceae and the pattern observed within the genus *Pseudomonas* reinforce the importance of microaeration for establishing populations with facultative aerobic metabolism. *Pseudomonas* was detected only in R2 with ethanol, indicating that, despite the potential capacity to degrade LAS, the low complexity of the substrate (ethanol) and the lower availability of aromatic structural carbon did not favor its activity. However, the inhibition of this genus in R2 with sucrose may be due to competition with other microorganisms adapted to the microaerobic regime and to the accumulation of metabolites that inhibit *Pseudomonas* growth.

The predominance of *Synergistaceae* and *Anaerolineaceae*, associated with the phyla *Synergistota* and *Chloroflexi*, respectively, in R1_S suggests the involvement of fermentative and syntrophic pathways during intermediate stages of anaerobic degradation. Although these groups participate in the fermentation of amino acids, organic acids, and other complex compounds, they are not directly involved in the initial degradation of LAS. *Synergistota* has been frequently reported in anaerobic systems treating LAS-containing effluents and as an efficient degrader of toxic compounds,^6^ although the corresponding enzymatic pathways may not always be active. Together with the enrichment of *Bacteroidota*, which degrades polymers and high-molecular-weight compounds^12^ but showed similar abundances in R1 and R2, these results indicate that sucrose favored a more diverse but functionally less specialized community for surfactant degradation. In contrast, the inhibition of *Actinomycetota*, with *Atopobiaceae* detected only in the inoculum, suggests sensitivity to LAS and microaeration.

The predominance of *Methanobacterium* (family *Methanobacteriaceae*, class *Methanobacteria*, phylum *Methanobacteriota*) across all systems reflects the maintenance of hydrogenotrophic methanogenesis, which is essential for H_2_ consumption and redox balance.^50^ However, the abundance of this genus was reduced in relation to the inoculum, especially with sucrose, suggesting that the accumulation of oxygenated or sulfonated intermediates from the partial degradation of LAS exerted competitive inhibition on the archaea.

Nevertheless, the greater presence of *Methanobacterium* in the microaerated reactors (R2) indicates that microaeration did not compromise methanogenesis but allowed functional coexistence between methanogenic populations and oxidative bacteria. This interaction is consistent with greater LAS removal in R2 with sucrose, where archaea likely participated in the final mineralization of carbon from the initial oxidative degradation of the surfactant.

Overall, the observed taxonomic pattern indicates that microaeration and sucrose acted as complementary selective forces, reducing diversity but promoting a functionally specialized community for LAS degradation. While R1 maintained predominantly fermentative populations (*Clostridium*, *Carnobacterium*, *Synergistaceae*, *Anaerolineaceae*), R2, with microaeration and sucrose, favored the establishment of oxidative and syntrophic consortia (*Pleomorphomonas*, *Desulfovibrio*, *Methanobacterium*), potentially associated with partial surfactant transformation and subsequent mineralization. The coexistence of these groups suggests a potential metabolic sequence, in which the initial transformation of LAS under microaerobic conditions may be followed by the anaerobic conversion of intermediates, resulting in the greater efficiency observed in R2_S.

Before discussing the microbial and functional responses observed in each reactor, it is important to note that LAS removal was evaluated based on dissolved-phase concentrations. Therefore, the observed decrease in LAS may result from a combination of biodegradation and biosorption mechanisms, since surfactants can be partially retained within the reactor through adsorption onto sludge particles and extracellular polymeric substances. Although the relative contribution of these mechanisms was not directly quantified in the present study, the microbiological and functional analyses revealed substantial shifts in microbial composition and metabolic potential associated with LAS transformation. These findings suggest that biological conversion likely contributed most significantly to the observed LAS removal, although a complete mass balance between soluble, adsorbed, and transformed fractions was beyond the scope of this work. Future studies should quantify the LAS associated with the sludge phase to better distinguish the relative contributions of biosorption and biodegradation.

#### 3.4.3 Intracellular Metabolism from Different Substrates Used under Strictly Anaerobic and Microaerobic Conditions

Anaerobic LAS biodegradation has been associated with a sequence of metabolic processes involving fumarate addition to the alkyl chain, β-oxidation, aromatic ring cleavage, and desulfonation.^10^ To investigate how the operational conditions may have affected these processes, the functional potential of the microbial communities was inferred from the 16S rRNA gene data using PICRUSt2. The relative abundance of predicted KEGG Orthologs (KOs) was then compared among the inoculum and the biomass collected from each reactor (Figure 6).

**Figure 6:**
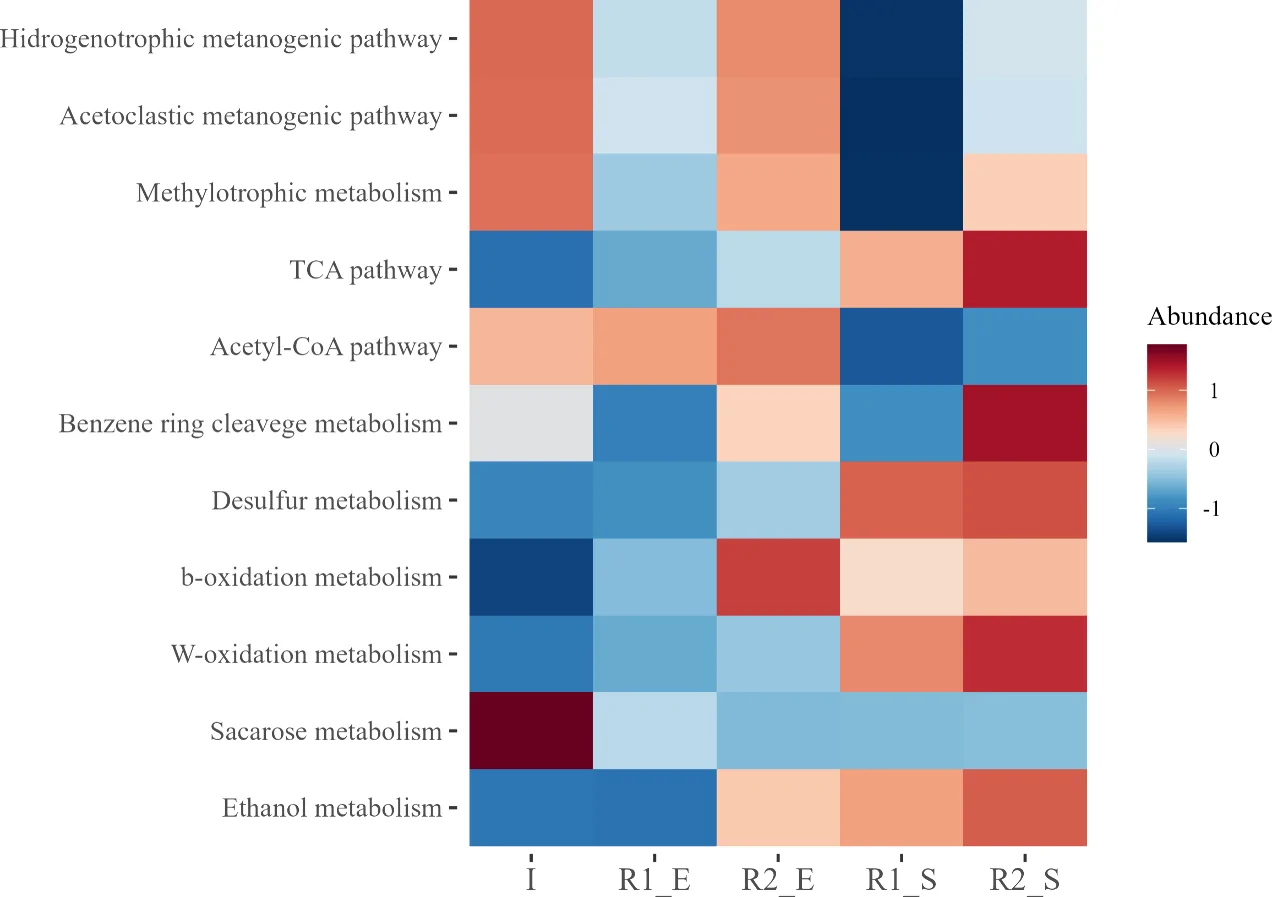
Z-score of the relative abundance of metabolic pathways involved in the biological degradation process of LAS.

The z-score normalization used in the heatmap represents the relative abundance of each predicted pathway across the samples. Positive values indicate that a pathway was comparatively more abundant in a given sample, whereas negative values indicate a lower relative abundance. Therefore, these results represent differences in predicted functional potential rather than direct measurements of gene expression or enzymatic activity.

The inoculum exhibited a broad functional profile, including pathways associated with sucrose metabolism, acetoclastic and hydrogenotrophic methanogenesis, methylotrophic metabolism, and Acetyl-CoA conversion. This pattern is consistent with a metabolically diverse anaerobic community not yet subjected to the selective pressures imposed by LAS, co-substrate type, and microaeration.^38^

Under ethanol feeding, both reactors were primarily characterized by pathways associated with methanogenesis and central carbon metabolism. R1_E showed a comparatively higher representation of the Acetyl-CoA pathway, whereas R2_E exhibited a stronger relative contribution of β-oxidation and hydrogenotrophic methanogenesis. These differences suggest that microaeration modified the predicted functional organization of the community, although this change did not result in a statistically significant improvement in LAS removal. Ethanol metabolism may have preferentially directed carbon and electron flow toward acetogenic and methanogenic processes, limiting the contribution of additional oxidative reactions to surfactant transformation.^6^

A more pronounced functional shift was observed after sucrose addition. R1_S showed a higher relative representation of sulfur metabolism, ω-oxidation, ethanol metabolism, and the tricarboxylic acid cycle. In R2_S, pathways associated with the tricarboxylic acid cycle, benzene ring cleavage, ω-oxidation, desulfurization, and ethanol metabolism were simultaneously enriched. This broader distribution of pathways potentially associated with the transformation of the alkyl chain, aromatic ring, and sulfonate group coincided with the highest LAS removal observed in the study.^12,46,47^

Overall, the predicted profiles indicate that ethanol maintained a functional structure more strongly associated with acetogenic and methanogenic metabolism, whereas sucrose promoted pathways potentially associated with the transformation of LAS and its intermediates. This effect was most evident under microaeration, suggesting that the combination of sucrose and limited oxygen availability favored a more integrated functional potential for LAS degradation.^51^ The specific microbial groups and KOs associated with these pathways are examined in the following subsections.

#### 3.4.4 Gene-Enzyme Linkage Analysis and Correlation between Microbial Community Structure and Functional Genes Involved in Different Metabolisms

##### 3.4.4.1 Methanogenic Acetoclastic, Hydrogenotrophic, and Methylotrophic Pathways

The predicted acetoclastic, hydrogenotrophic, and methylotrophic methanogenic pathways were examined to evaluate how co-substrate type and microaeration influenced carbon conversion during LAS degradation (Figure 7). Across all reactors, the majority of predicted methanogenic functions were associated with the genus *Methanobacterium*, indicating that hydrogenotrophic methanogenesis represented the predominant methane-producing route under the evaluated conditions. This predominance was more evident in the ethanol-fed reactors, consistent with the availability of reducing equivalents generated during ethanol oxidation.

**Figure 7:**
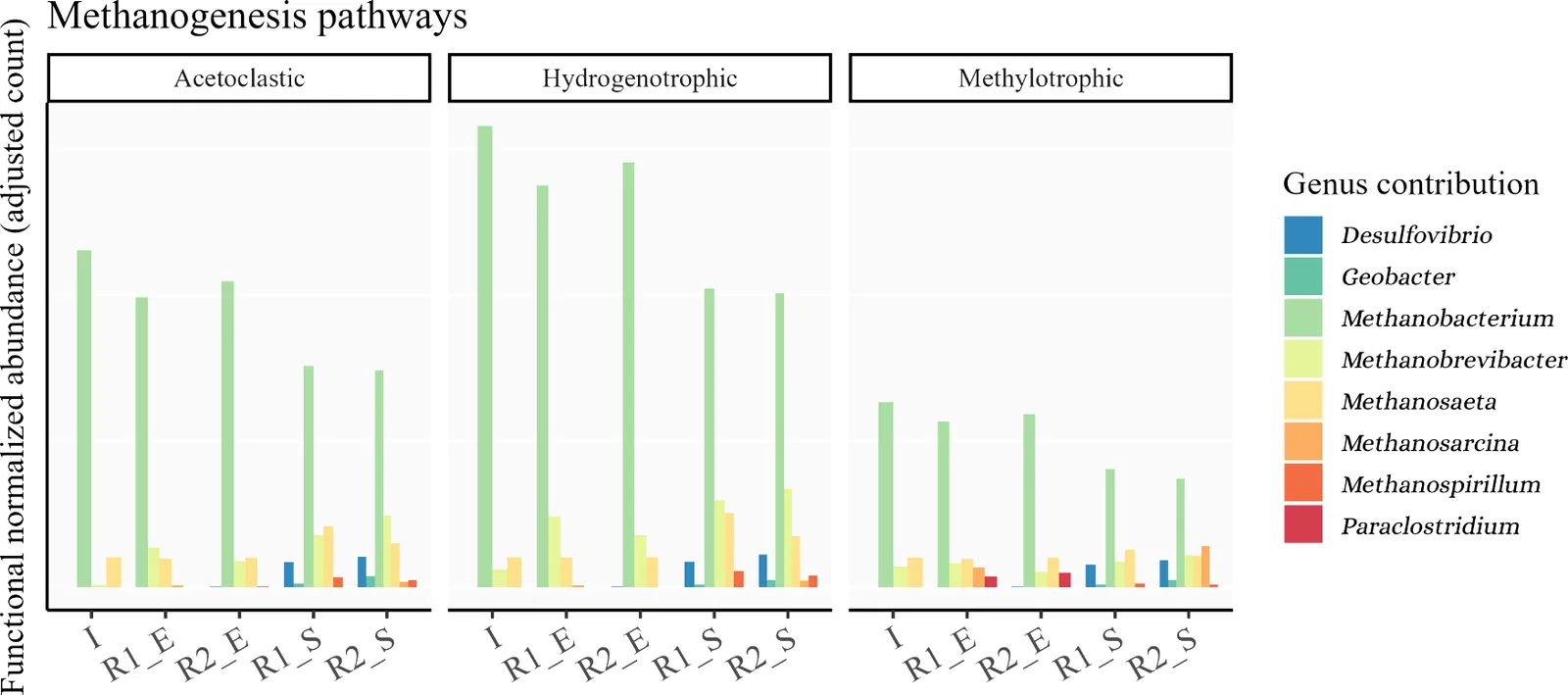
Contribution of the most significant genus in methanogenic pathways.

In the acetoclastic pathway, the main predicted KOs were K00197, K01895, K00925, and K03388 (Figure S4), which are associated with enzymes involved in acetate activation and conversion to methane.^52,53^ Ethanol-fed reactors exhibited a greater overall abundance of these predicted functions, particularly R2_E, where K01895 became more abundant than K00197. In contrast, sucrose-fed reactors showed lower representation of acetoclastic KOs, although K01895 remained the predominant ortholog, followed by K00925 and K03388. This shift suggests that sucrose supplementation reduced the relative contribution of acetoclastic methanogenesis while favoring alternative metabolic processes involved in carbon transformation.

A similar trend was observed for the hydrogenotrophic pathway. Ethanol-fed reactors showed a higher abundance of predicted hydrogenotrophic KOs, with K11261, encoding a ferredoxin-dependent hydrogenase,^54^ predominating in all systems except R2_S. In the microaerated sucrose reactor, K03388, K03389, and K03390 became the dominant orthologs, indicating a redistribution of the predicted methanogenic functions under conditions that also favored oxidative pathways associated with LAS transformation.

The methylotrophic pathway represented the smallest proportion of the predicted methanogenic functions in all reactors. Nevertheless, K03388, K03389, and K03390, which encode subunits of methyl-coenzyme M reductase,^49,55^ were consistently detected. These orthologs were comparatively more abundant under ethanol feeding, whereas sucrose promoted a more balanced distribution, indicating that methylotrophic methanogenesis remained active but represented a smaller fraction of the overall methanogenic potential.

Besides *Methanobacterium*, the predicted methanogenic pathways were also associated with *Methanobrevibacter*, *Methanosaeta*, and *Methanosarcina*. *Methanobrevibacter* was mainly related to hydrogenotrophic methanogenesis, whereas *Methanosaeta* and *Methanosarcina* are classical acetoclastic methanogens, with the latter also capable of methylotrophic metabolism.^48^ The coexistence of these genera indicates that multiple methanogenic routes remained potentially active regardless of the operational condition.

Overall, ethanol promoted a greater predicted abundance of methanogenic functions, whereas sucrose, particularly under microaeration, shifted the predicted metabolic profile toward a lower relative contribution of methanogenesis. This pattern is consistent with the broader redistribution of predicted metabolic functions observed in R2_S, where pathways associated with LAS transformation became proportionally more represented. The specific oxidative pathways potentially responsible for this functional shift are discussed in the following sections.

##### 3.4.4.2 TCA and Acetyl-CoA Pathways and Benzene Ring Cleavage Metabolism

To evaluate how co-substrate type and microaeration influenced central carbon metabolism and aromatic compound transformation, the predicted TCA cycle, Acetyl-CoA, and benzene ring cleavage pathways were analyzed (Figure 8). The systems fed with ethanol showed a predominance of *Methanobacterium* and *Pseudomonas*, with the former being more abundant in the conventional UASB and the latter in the microaerated system, suggesting that the controlled presence of oxygen favored microorganisms with more oxidative metabolism. With sucrose, the behavior was distinct between the systems. In R1, *Tolumonas* and *Pleomorphomonas* stood out, while in R2 *Aeromonas* and *Desulfovibrio* prevailed, indicating greater variation in microbial structure under the different operating conditions.

**Figure 8:**
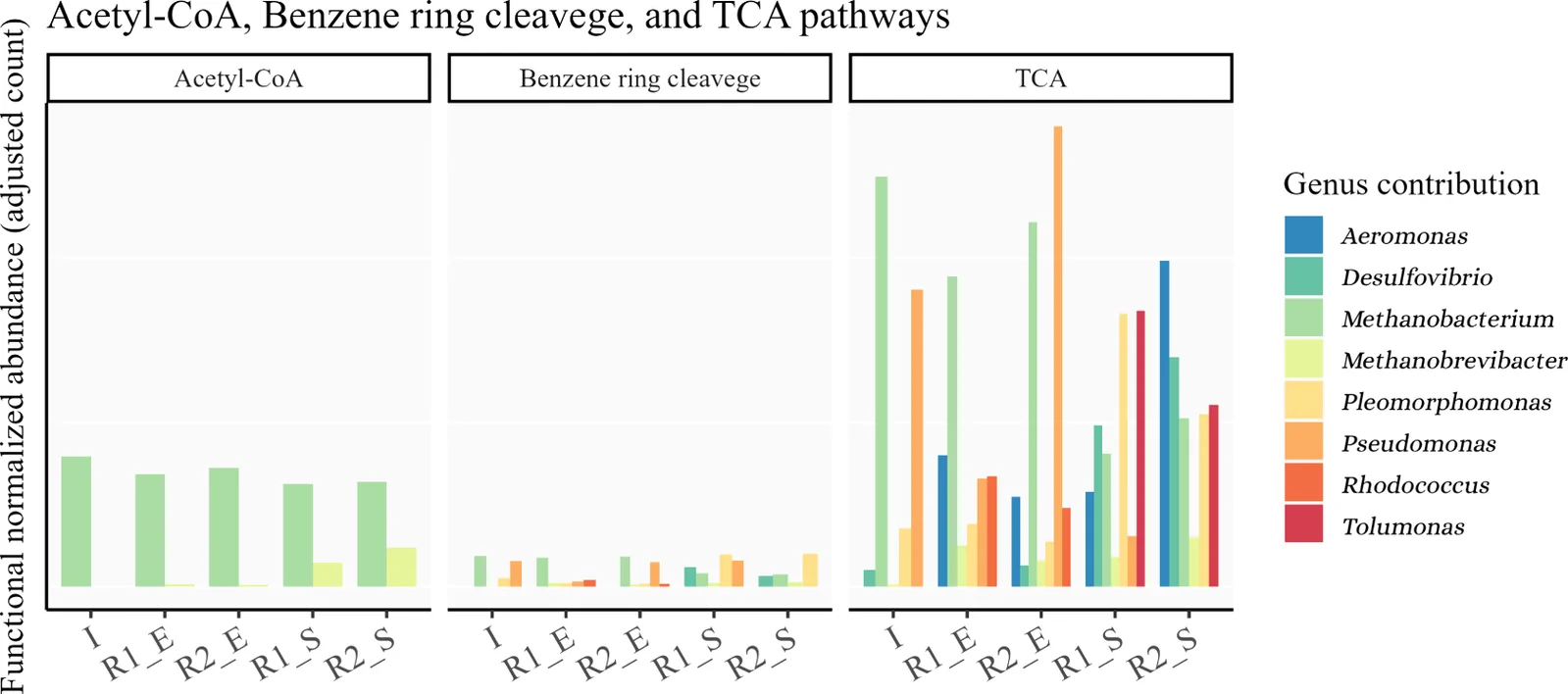
Contribution of the most significant genus to the TCA, Acetyl-CoA, and benzene ring cleavage metabolism pathways.

In general, the microaerated systems presented a greater number of KO in this pathway, suggesting greater metabolic activity associated with the intermediate oxidation of organic compounds (Figure S5). In terms of KO abundance, reactors with ethanol showed higher values than those with sucrose, with K00247 being the most representative, followed by K00174 and K00175. In systems with sucrose, the pattern was reversed, with K00175 being the most abundant, followed by K00174 and K00247.

In the benzene ring cleavage pathway, reactors fed with sucrose showed higher KO abundance, indicating greater potential for the degradation of aromatic compounds under this condition. Among ethanol-associated genera, the most common was *Methanobacterium*. At the same time, with sucrose, *Pleomorphomonas* stood out, both of which have been associated with aromatic compound transformation and the processing of recalcitrant byproducts, possibly derived from LAS.^46^ In all systems, the main KO identified were K03388 and K07104, with the R2_E reactor standing out, highlighting the importance of these routes and suggesting a relevant role for microaeration combined with ethanol in activating oxidative pathways complementary to surfactant degradation.

In the Acetyl-CoA pathway, similar behavior was observed across the systems, with *Methanobacterium* as the main genus, although the reactors with sucrose showed greater total abundance, especially R2_S, which also showed an increase in *Methanobrevibacter*. This predominance reinforces the importance of these archaea in the final conversion of intermediates into methane, especially via the acetoclastic pathway. In all reactors except R2_S, K00197 was the most abundant KO, followed by K00194, which represent central steps in acetate activation.^49,51^ Among the systems, R1_E showed the highest KO abundance. At the same time, R2_S had the lowest, suggesting that ethanol favored greater metabolic flux via Acetyl-CoA and, consequently, greater energy efficiency and LAS degradation. In contrast, sucrose partially limited this route, possibly by promoting the accumulation of fermentative intermediates.

##### 3.4.4.3 Desulfur Metabolism

To investigate the potential role of sulfur metabolism in LAS degradation under the different operational conditions, the predicted sulfur metabolism pathway was analyzed (Figure 9). In taxonomic and functional terms, the systems with sucrose showed a profile that favors the reduction of the sulfonate group. *Desulfovibrio* and *Propionivibrio* (R1_S and R2_S) were the dominant genera, with R2_S showing the highest overall abundance of microorganisms associated with this route. The high abundance of *Desulfovibrio* is mechanistically relevant, as these sulfate-reducing bacteria perform the final steps of sulfite/sulfonate reduction to sulfide, releasing the organic skeleton for subsequent anaerobic mineralization.^38,55^ This may help explain why the sucrose-containing systems, particularly R2_S, exhibited greater LAS removal. That is, active desulfonation makes LAS intermediates more accessible to subsequent catabolism.

**Figure 9:**
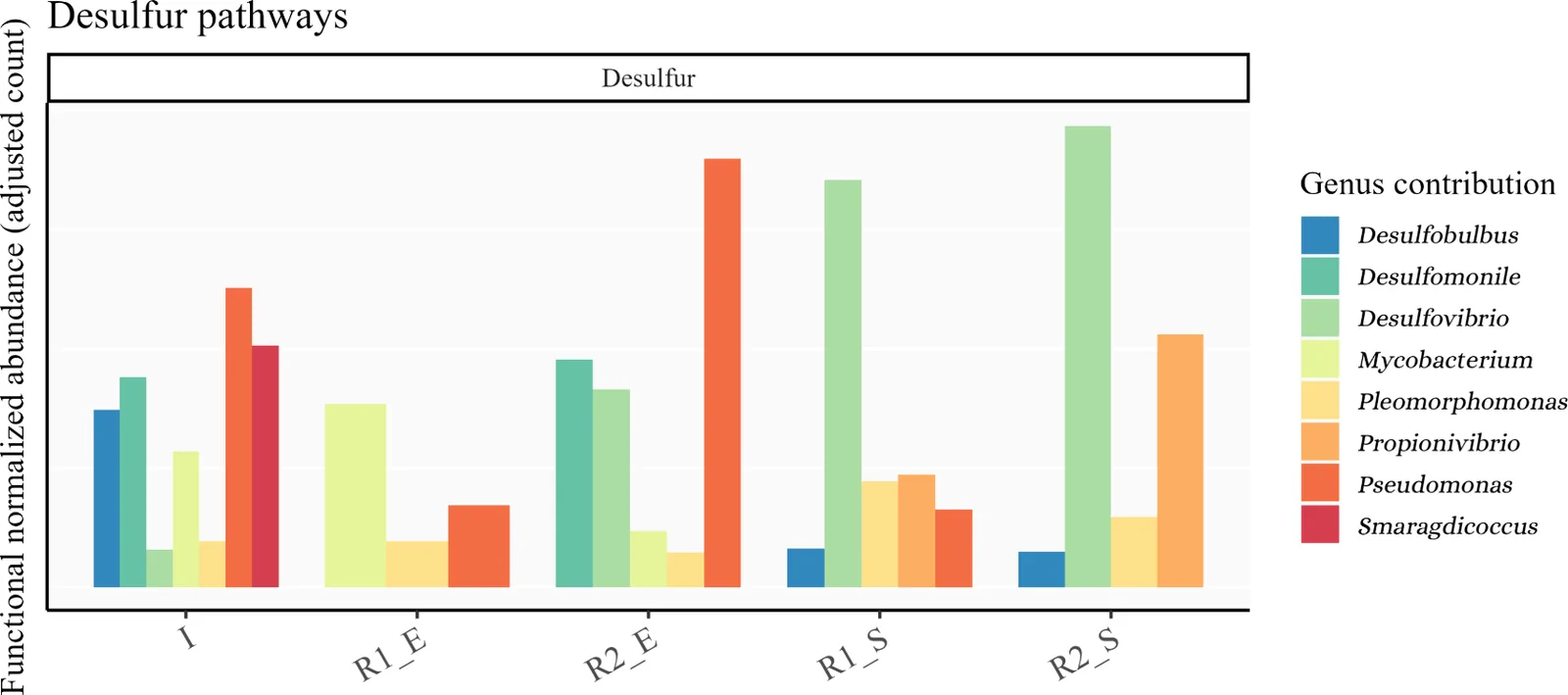
Contribution of the most significant genus to desulfur metabolism.

As for KO, K00390 (ferredoxin-dependent sulfite reductase) stood out in all systems, especially in the sucrose-containing reactors (Figure S6). The high level of K00390 in R2_S and R1_S suggests a greater predicted potential to convert sulfite into sulfide, a reaction potentially involved in LAS desulfonation and corroborates the functional importance of this pathway for surfactant removal.^56^ Furthermore, R1_S showed the highest total abundance of sulfur metabolism KO genes, suggesting a strong overall enzymatic potential for desulfonation even in the absence of microaeration.

However, the high identification and/or inference of metabolic functionality based on the number of KO does not guarantee the removal efficiency of LAS. The difference between R1_S and R2_S assumes that desulfonation needs to be coupled with initial oxidative steps for LAS mineralization to be effective. Wei et al.^57^ and Yadav et al.^58^ point out that alkyl chain cleavage and aromatic ring opening precede or coincide with desulfonation.

In R1_S, despite many KO inferred and identified (including K00390), the absence of oxidative support limited by strict anaerobiosis restricts the generation of intermediates that sulfate-reducing microorganisms can fully transform, explaining why R1_S had lower removal than R2_S. In contrast, R2_S combines a high presence of sulfur-reducing microorganisms with oxidative microzones induced by microaeration, which promotes both the necessary partial oxidation and subsequent desulfonation, resulting in the highest removal observed. In the ethanol systems, the profile identified was different. There was a greater balance between K00299 (relating to the enzyme Adenylyl-sulfate reductase), K00390, and K04091 (related to the enzyme cysteine desulfurase), and the communities included *Pseudomonas*, *Mycobacterium*, and *Desulfomonile* (R2_E). This enzymatic composition indicates that both sulfate activation (K00299), sulfite reduction (K00390), and sulfur transfer (K04091) were present.^56^ Even so, LAS removal did not show a statistically significant difference between R1_E and R2_E. This likely occurred due to carbon competition and metabolic prioritization, which limit the impact on LAS removal.

##### 3.4.4.4 LAS Degradation by ω- and β-Oxidation Metabolism

To evaluate the influence of co-substrate type and microaeration on LAS side-chain degradation, the predicted ω-oxidation and β-oxidation pathways were analyzed (Figure 10), complementing the ring cleavage and desulfonation routes discussed previously, where the former is responsible for activation and the latter for sequential degradation.^10,59^

**Figure 10:**
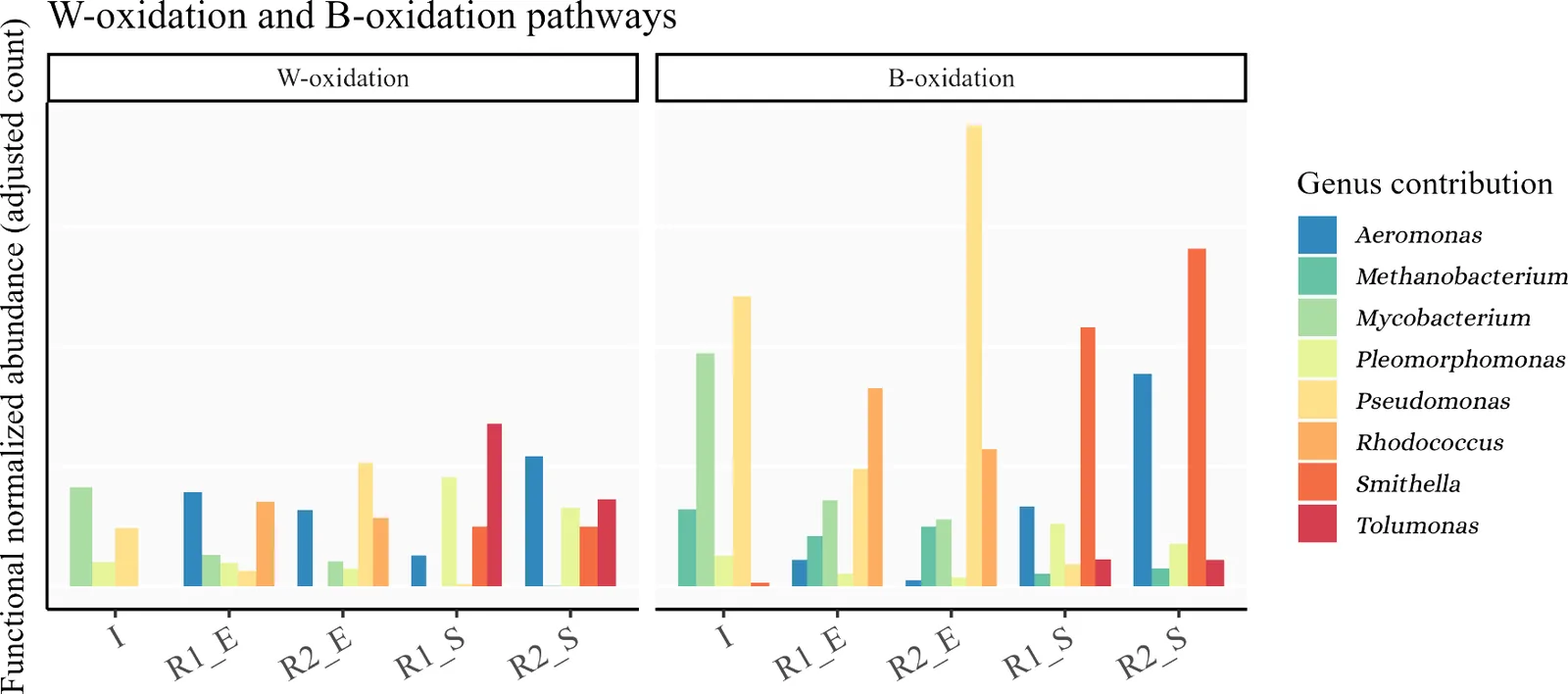
Contribution of the most significant genus to the ω-oxidation and β-oxidation pathways.

β-oxidation showed higher predicted abundance than ω-oxidation in all systems, consistent with the progressive shortening of the LAS alkyl chain by successive removal of two-carbon units.^49,60^ The greatest abundance of both pathways occurred in R2_S, coinciding with the highest LAS removal and suggesting that microaeration favored the coupling between initial activation and subsequent side-chain degradation.

In the ethanol-fed reactors, ω-oxidation was mainly associated with Aeromonas and Rhodococcus in R1_E, whereas Pseudomonas became predominant in R2_E, consistent with its recognized ability to initiate the oxidation of hydrocarbons and anionic surfactants.^55^ The predominance of K00128 and K00121 (Figure S7), encoding NAD^+^-dependent alcohol dehydrogenase and aldehyde dehydrogenase,^61^ suggests activation of the terminal alkyl chain. However, ethanol likely acted as the preferred electron donor, limiting the coupling of these initial reactions with downstream degradation and explaining the absence of significant differences in LAS removal between R1_E and R2_E.

Under sucrose, the oxidative profile shifted from K00128 toward K00239, K00240, and K00241, encoding acyl-CoA dehydrogenases and enoyl-CoA hydratases involved in β-oxidation,^61,62^ suggesting a greater predicted potential for side-chain degradation following the initial oxidation step. This metabolic shift was accompanied by changes in the dominant genera, with Tolumonas and Pleomorphomonas prevailing in R1_S and Aeromonas and Tolumonas in R2_S, consistent with the enrichment of microorganisms associated with long-chain hydrocarbon oxidation and aromatic compound degradation.

The β-oxidation profile supported this interpretation. In the ethanol-fed reactors, Pleomorphomonas and Pseudomonas predominated together with K00074 (acetyl-CoA acetyltransferase) and K01897 (3-hydroxyacyl-CoA dehydrogenase), although the metabolic preference for ethanol probably limited the conversion of this functional potential into higher LAS removal. In contrast, sucrose favored the enrichment of Smithella and Aeromonas, while K01897 was particularly abundant in R1_S, indicating active conversion of 3-hydroxyacyl-CoA to β-ketoacyl-CoA.^57^ Despite the higher abundance of this KO in R1_S, R2_S achieved the greatest LAS removal, suggesting that microaeration enhanced the integration of ω- and β-oxidation and promoted more efficient degradation of partially oxidized intermediates.

##### 3.4.4.5 Ethanol and Sucrose Metabolism

The predicted pathways associated with ethanol and sucrose metabolism were examined to determine how each co-substrate shaped carbon conversion and its potential interaction with LAS degradation (Figure 11). In the ethanol-fed reactors, *Pseudomonas* and *Methanobacterium* were the predominant genera associated with ethanol metabolism, indicating the coexistence of bacteria potentially involved in ethanol oxidation and hydrogenotrophic methanogens capable of consuming the resulting intermediates.

**Figure 11:**
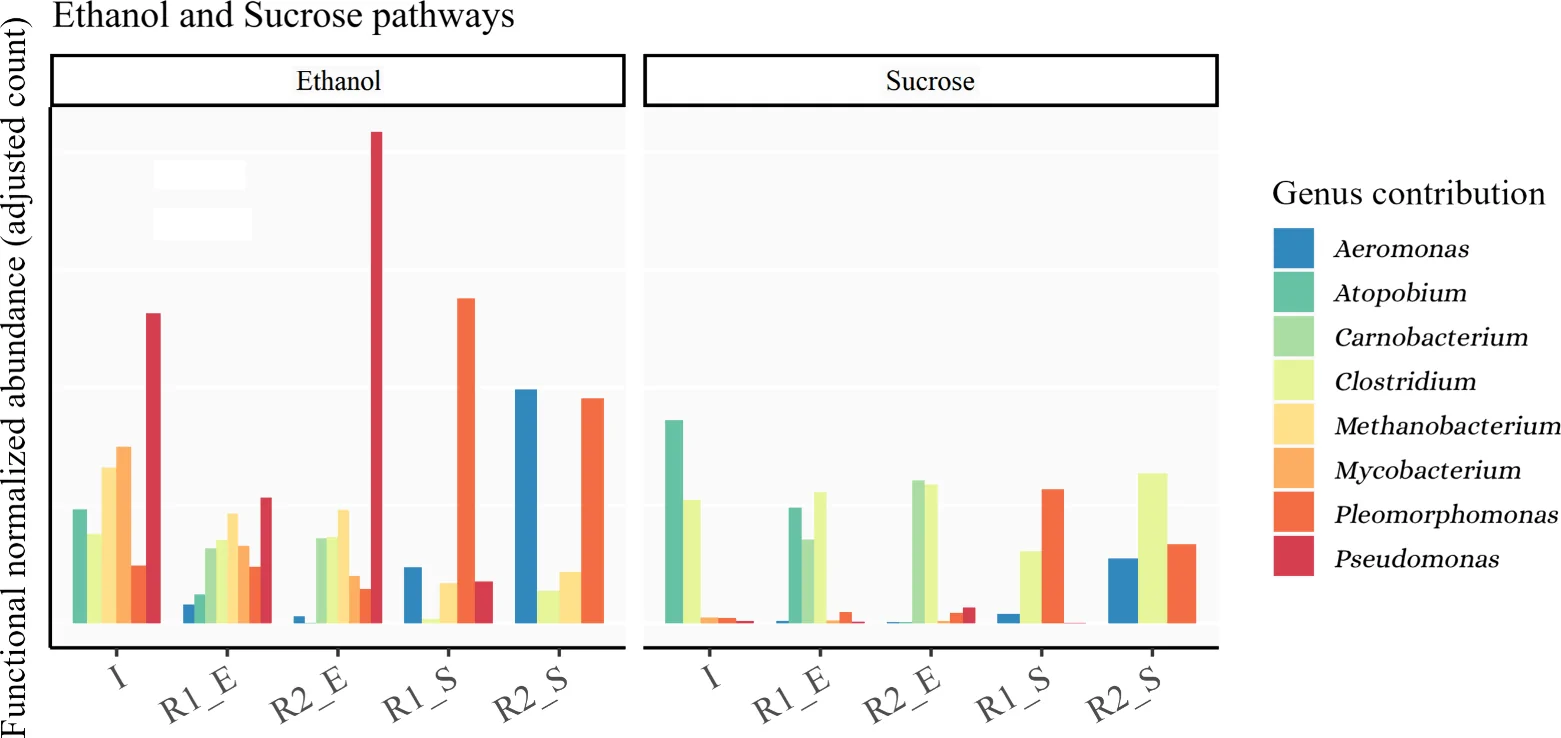
Contribution of the most significant genus to the ethanol and sucrose pathways.

The main predicted KOs associated with this pathway were K01895, related to acetaldehyde dehydrogenase and the conversion of acetaldehyde into Acetyl-CoA, and K01897, related to 3-hydroxyacyl-CoA dehydrogenase and β-oxidation (Figure S8). The greater relative abundance of K01897 in the ethanol-fed reactors suggests that carbon metabolism was strongly directed toward the conversion of ethanol and its intermediates. Because ethanol is readily metabolized, its preferential consumption may have supported microbial growth and methanogenic metabolism while limiting the relative contribution of LAS as a carbon or electron source.

Predicted functions associated with ethanol metabolism were also detected in the sucrose-fed reactors, indicating metabolic connectivity between sugar fermentation and pathways involving ethanol-derived intermediates. Under these conditions, *Pleomorphomonas* predominated in R1_S, whereas Aeromonas was more strongly associated with R2_S. The relative abundance pattern of the KOs was also reversed, with K01895 exceeding K01897, suggesting a greater potential for the conversion of fermentative intermediates into Acetyl-CoA and a comparatively lower representation of β-oxidation-related functions within this specific pathway. Although R1_S exhibited the highest total abundance of KOs associated with ethanol metabolism, R2_S achieved greater LAS removal, indicating that the abundance of an individual pathway alone was insufficient to explain reactor performance.

The sucrose metabolism pathway further distinguished the microbial responses to the two co-substrates. Even in R1_E and R2_E, where sucrose was not supplied, functions related to sucrose metabolism were detected at a basal level, possibly reflecting the broad carbohydrate-processing potential of the microbial community. *Clostridium* was the predominant genus in these reactors, accompanied by Atopobium in R1_E and *Carnobacterium* in R2_E. These fermentative taxa may contribute to the production of organic acids that subsequently support acetogenic and methanogenic populations. Consistently, K01193, associated with sucrose phosphorylase, was comparatively more abundant under ethanol feeding, indicating the predicted occurrence of carbohydrate–conversion routes capable of generating glucose-1-phosphate under reducing conditions.

When sucrose was directly supplied, the functional and taxonomic profiles changed markedly. *Pleomorphomonas* and *Clostridium* predominated in R1_S, whereas their relative contributions varied in R2_S, evidencing a restructuring of the community in response to the carbohydrate co-substrate. In both sucrose-fed reactors, K01187, associated with invertase or β-fructofuranosidase, became the most abundant predicted KO in this pathway. This enzyme catalyzes the initial hydrolysis of sucrose into glucose and fructose, thereby supplying intermediates to glycolysis, the TCA cycle, and Acetyl-CoA formation.

Therefore, the predominance of K01187 indicates a greater predicted capacity for the direct fermentation of sucrose and the generation of central metabolic intermediates. These intermediates may have supported methanogenic carbon conversion while also sustaining microbial populations potentially involved in LAS transformation. However, because R2_S achieved the highest LAS removal despite not consistently exhibiting the greatest abundance of every co-substrate-related KO, the results indicate that reactor performance was more closely associated with the integration of fermentative and oxidative metabolic potentials than with the isolated abundance of the ethanol or sucrose pathways.

##### 3.4.4.6 Impact of Microaeration and Co-substrate on LAS Metabolic Pathways

To integrate the microbiological and functional findings obtained throughout this study, the combined effects of microaeration and co-substrate on the predicted LAS degradation pathways are discussed below. The results indicate that the influence of microaeration on LAS degradation was strongly associated with the metabolic environment established by the co-substrate. Although oxygen supplementation was applied under the same operational conditions, its effects differed substantially between ethanol- and sucrose-fed reactors, suggesting that the response to microaeration was governed not only by oxygen availability but also by the structure and functionality of the microbial consortium supported by each carbon source.

Under ethanol supplementation, LAS degradation appeared to be primarily driven by conventional anaerobic processes. The predominance of fermentative and methanogenic microorganisms, together with the enrichment of pathways associated with carbon fermentation and methane production, suggests that most of the electron flow was likely directed toward the conversion of readily biodegradable substrates. Consequently, microaeration produced only limited changes in LAS removal, indicating that the oxygen supplied was insufficient to substantially redirect the dominant metabolic routes established in the reactor.

In contrast, sucrose supplementation resulted in a markedly different metabolic response. The greater availability of fermentative intermediates likely created conditions that favored syntrophic interactions and increased metabolic flexibility, allowing the microbial community to more effectively exploit the oxygen introduced through microaeration. This shift was accompanied by the enrichment of predicted functional pathways potentially associated with aromatic compound transformation, side-chain oxidation, sulfur metabolism, and the subsequent conversion of degradation intermediates. Collectively, these predictions are consistent with a greater potential for LAS transformation and further processing of its degradation products.

These findings suggest that microaeration may not have simply increased the availability of an additional electron acceptor, but may also have influenced the sequence of metabolic reactions potentially involved in LAS degradation. Under sucrose supplementation, oxygen may have facilitated the initial activation of the surfactant molecule and the transformation of aromatic intermediates that are typically degraded slowly under strictly anaerobic conditions. The resulting intermediates could subsequently be utilized by anaerobic microorganisms through pathways potentially involving β-oxidation, sulfur transformation, and methanogenesis, thereby establishing a complementary interaction between oxidative and reductive processes.

Therefore, the combined application of microaeration and sucrose appears to have favored a hybrid degradation pathway in which oxygen-dependent reactions may have enhanced the initial transformation of LAS, while anaerobic metabolism likely contributed to the subsequent conversion of intermediates. This metabolic coupling provides a plausible mechanistic explanation for the superior LAS removal observed in the microaerated sucrose-fed reactor and highlights the importance of simultaneously considering redox conditions and co-substrate selection when designing strategies to improve surfactant removal in anaerobic wastewater treatment systems.

A summary comparing the main findings of the present study with those of previous studies discussed throughout the manuscript is provided in Table S1 of the Supplementary Material.

## 4 Conclusions

This study demonstrated the potential of microaeration as a strategy to improve LAS degradation under anaerobic conditions. However, the response to oxygen supplementation differed according to the co-substrate employed, suggesting that the metabolic environment established within the reactor may influence the extent to which microaeration stimulates surfactant transformation. In this regard, the most pronounced improvement in LAS removal was observed when sucrose was used as the co-substrate.

The enhanced performance achieved under sucrose supplementation was associated with a substantial restructuring of the microbial community and its metabolic functions. Microaeration favored the establishment of a more specialized consortium capable of coupling oxidative and anaerobic degradation pathways, thereby facilitating the conversion of aromatic and sulfonated intermediates while preserving the overall functionality of the anaerobic process. These findings suggest that the benefits of microaeration extend beyond direct oxygen supply and are closely linked to its ability to reshape microbial metabolism and promote complementary degradation routes.

From a practical standpoint, the results suggest that microaeration can be implemented as a low-cost process intensification strategy to improve LAS removal in UASB reactors treating domestic wastewater. The positive response observed under sucrose supplementation indicates that oxygen supply and co-substrate selection may act synergistically to overcome metabolic limitations associated with anaerobic LAS degradation. Because the improvement was achieved under an HRT typical of full-scale UASB reactors, the proposed approach may represent a feasible alternative for enhancing surfactant removal in existing treatment plants without substantial infrastructural modifications. Future studies should investigate the effects of different microaeration rates, redox mediator supplementation, alternative electron acceptors such as nitrate and nitrite, evaluate the integration of bioaugmentation or co-metabolic strategies, and assess the long-term impacts of microaeration on effluent quality under real-scale operating conditions.

## Supplementary Material



## Notes

Schematic representations of the continuous experimental set-up and batch experiment; LAS removal during the batch experiment; predicted genes associated with acetoclastic, hydrogenotrophic, and methylotrophic methanogenic pathways; predicted genes associated with acetyl-CoA metabolism, benzene ring cleavage, and TCA pathways; predicted genes associated with sulfur metabolism, ω-oxidation, β-oxidation, ethanol metabolism, and sucrose metabolism; comparison of the present study with previous studies regarding LAS removal, microaeration, co-substrate selection, biogas production, and microbial community structure. (PDF)

## References

[1] Mariraj
Mohan S., Swathi T. (2022). A review on upflow anaerobic sludge
blanket reactor: Factors affecting performance, modification of configuration
and its derivatives. Water Environ. Res..

[2] Terreros-Mecalco J., Velazquez-Garduño G., Archundia Velarde E.D., Hernández Ruiz P.E. (2024). Effect of lactose
as a co-substrate
in the biodegradation of an anionic surfactant “LAS”
by anaerobic digestion. Biocatal. Agric. Biotechnol..

[3] Motteran F., Varesche M. B. A., Lara-Martin P.A. (2022). Assessment
of the aerobic and anaerobic
biodegradation of contaminants of emerging concern in sludge using
batch reactors. Environ. Sci. Pollut. Res. 2022
2956.

[4] Villabona-Ortíz A., Tejada-Tovar C., De-La-Rosa-Jiménez L. (2021). Evaluation of Parameters
in the Removal of Linear Alkylbenzene Sulfonate Anionic Surfactant
Using Electrocoagulation. Rev. Fac. Ing..

[5] Mecalco J.T., Hilario J.E., Faviel S.P. (2024). Simultaneous
Biodegradation of Linear
Alkylbenzensulfonate in Anaerobic Reactors. Two Stages, Water, Air, Soil Pollut. 2024 2358.

[6] Motteran F., Nadai B.M., Braga J.K., Silva E.L., Varesche M. B. A. (2018). Metabolic
routes involved in the removal of linear alkylbenzene sulfonate (LAS)
employing linear alcohol ethoxylated and ethanol as co-substrates
in enlarged scale fluidized bed reactor. Sci.
Total Environ..

[7] Teran F. J. C., Santos R.R., Silva Y.L., Cuba R. M. F., Silveira J.R. (2022). Linear Alkylbenzene
Sulfonate Removal by Oxidative Ozonation Coupled with UV/H2O2 Oxidative
Process. Eur. J. Eng. Technol. Res..

[8] Okada D.Y., Esteves A.S., Delforno T.P., Hirasawa J.S., Duarte I. C. S., Varesche M. B. A. (2013). Influence of
co-substrates in the
anaerobic degradation of an anionic surfactant. Brazilian J. Chem. Eng..

[9] Costa J.L., Silva L.G., Veras S. T. S., Gavazza S., Florencio L., Motteran F., Kato M.T. (2025). Use of nitrate,
sulphate, and iron
(III) as electron acceptors to improve the anaerobic degradation of
linear alkylbenzene sulfonate: effects on removal potential and microbiota
diversification. Environ. Sci. Pollut. Res.
2024 3228.

[10] Lara-Martín P.A., Gómez-Parra A., Sanz J.L., González-Mazo E. (2010). Anaerobic
Degradation Pathway of Linear Alkylbenzene Sulfonates (LAS) in Sulfate-Reducing
Marine Sediments. Environ. Sci. Technol..

[11] Unuofin J.O., Okoh A.I., Nwodo U.U. (2019). Recovery
of laccase-producing gammaproteobacteria
from wastewater. Biotechnol. Reports.

[12] Wei T., Ran T., Rong W., Zhou Y. (2024). Efficient and sustainable removal
of linear alkylbenzene sulfonate in a membrane biofilm: Oxygen supply
dosage impacts mineralization pathway. Water
Res. X.

[13] Li X., Wang Z., He Y., Wang Y., Wang S., Zheng Z., Wang S., Xu J., Cai Y., Ying H. (2024). A Comprehensive Review of the Strategies
to Improve Anaerobic Digestion:
Their Mechanism and Digestion Performance. Methane
2024, Vol 3, Pages 227-256.

[14] Siqueira J. P. S., Pereira A.M., Dutra A. M. M., Firmino P. I. M., dos
Santos A.B. (2018). Process bioengineering applied to BTEX degradation
in microaerobic treatment systems. J. Environ.
Manage..

[15] Buakaew T., Ratanatamskul C. (2024). Unveiling the influence of microaeration and sludge
recirculation on enhancement of pharmaceutical removal and microbial
community change of the novel anaerobic baffled biofilm - membrane
bioreactor in treating building wastewater. Sci. Total Environ..

[16] Carvalho J. R. S., Amaral F.M., Florencio L., Kato M.T., Delforno T.P., Gavazza S. (2020). Microaerated UASB reactor treating textile wastewater:
The core microbiome and removal of azo dye Direct Black 22. Chemosphere.

[17] do
Nascimento J.G. da S., de Araújo M. H.
P., dos Santos A.B., da Silva M. E. R., Firmino P. I. M. (2021). Can microaeration boost the biotransformation
of parabens in high-rate anaerobic systems?. Process Saf. Environ. Prot..

[18] Khadir A., Nakhla G., Karki R., Raskin L., Muller C., Guevarra K., Summers A., Pierce L., Shahbaz P., Bell K., Skerlos S., Bronstad E. (2025). Micro-aeration
for
hydrogen sulfide reduction in full-scale anaerobic digesters with
limited headspace: Performance and sulfide reduction kinetics. Process Saf. Environ. Prot..

[19] Zhou Y., Li R., Guo B., Zhang L., Zou X., Xia S., Liu Y. (2020). Greywater treatment using an oxygen-based
membrane biofilm reactor:
Formation of dynamic multifunctional biofilm for organics and nitrogen
removal. Chem. Eng. J..

[20] Zhou Y., Li R., Guo B., Xia S., Liu Y., Rittmann B.E. (2022). The influent
COD/N ratio controlled the linear alkylbenzene sulfonate biodegradation
and extracellular polymeric substances accumulation in an oxygen-based
membrane biofilm reactor. J. Hazard. Mater..

[21] Cui X., Ren Q., Zhang J., Zhou Y. (2022). Removal kinetics of linear alkylbenzene
sulfonate in a batch-operated oxygen based membrane biofilm reactor
treating greywater: Quantitative differentiation of adsorption and
biodegradation. Sci. Total Environ..

[22] Wei T., Ran T., Rong W., Zhou Y. (2024). Efficient and sustainable removal
of linear alkylbenzene sulfonate in a membrane biofilm: Oxygen supply
dosage impacts mineralization pathway. Water
Res. X.

[23] Ran T., Wei T., Xia L., Rong W., Zhou Y. (2024). Dissolved oxygen dependence
of Microbe-Derived dissolved organic nitrogen dynamics during membrane
biofilm-based linear alkylbenzene sulfonate mineralization. Chem. Eng. J..

[24] Liu Y., Dai A., Xia L., Zhou Y., Ren T., Huang Y., Zhou Y. (2024). Deciphering
the roles of nitrogen source in sharping synchronous
metabolic pathways of linear alkylbenzene sulfonate and nitrogen in
a membrane biofilm for treating greywater. Environ.
Res..

[25] Chernicharo C. A. L., van Lier J.B., Noyola A., Bressani Ribeiro T. (2015). Anaerobic
sewage treatment: state of the art, constraints and challenges. Rev. Environ. Sci. Bio/Technology 2015 144.

[26] Angelidaki I., Alves M., Bolzonella D., Borzacconi L., Campos J.L., Guwy A.J., Kalyuzhnyi S., Jenicek P., Van Lier J.B. (2009). Defining the biomethane potential
(BMP) of solid organic wastes and energy crops: a proposed protocol
for batch assays. Water Sci. Technol..

[27] Firmino P. I. M., da Silva M. E. R., Cervantes F.J., dos Santos A.B. (2010). Colour removal of dyes from synthetic and real textile
wastewaters in one- and two-stage anaerobic systems. Bioresour. Technol..

[28] APHA , Standard Methods for the Examination of Water and Waste Water, 22nd ed., 2012. https://www.scirp.org/reference/ReferencesPapers?ReferenceID=1982598 (accessed June 4, 2025).

[29] Silva L.G., Gavazza S., Florencio L., Kato M.T. (2017). Quantificação
de alquilbenzeno linear sulfonato em estação de tratamento
de efluentes e rios por cromatografia líquida de alta eficiência
e extração em fase sólida. Quim. Nova.

[30] Parada A.E., Needham D.M., Fuhrman J.A. (2016). Every base matters: Assessing small
subunit rRNA primers for marine microbiomes with mock communities,
time series and global field samples. Environ.
Microbiol..

[31] Caporaso J.G., Lauber C.L., Walters W.A., Berg-Lyons D., Huntley J., Fierer N., Owens S.M., Betley J., Fraser L., Bauer M., Gormley N., Gilbert J.A., Smith G., Knight R. (2012). Ultra-high-throughput microbial community
analysis on the Illumina HiSeq and MiSeq platforms. ISME J..

[32] Callahan B.J., McMurdie P.J., Rosen M.J., Han A.W., Johnson A. J. A., Holmes S.P. (2016). DADA2: High-resolution sample inference from Illumina
amplicon data. Nat. Methods 2016 137.

[33] Langille M. G. I., Zaneveld J., Caporaso J.G., McDonald D., Knights D., Reyes J.A., Clemente J.C., Burkepile D.E., Vega Thurber R.L., Knight R., Beiko R.G., Huttenhower C. (2013). Predictive
functional profiling of microbial communities using 16S rRNA marker
gene sequences. Nat. Biotechnol. 2013 319.

[34] Kanehisa M., Sato Y., Kawashima M., Furumichi M., Tanabe M. (2016). KEGG as a reference resource for
gene and protein annotation. Nucleic Acids Res..

[35] Kanehisa M., Goto S. (2000). KEGG: Kyoto Encyclopedia
of Genes and Genomes. Nucleic Acids Res..

[36] Douglas G.M., Maffei V.J., Zaneveld J.R., Yurgel S.N., Brown J.R., Taylor C.M., Huttenhower C., Langille M. G. I. (2020). PICRUSt2 for
prediction of metagenome functions. Nat. Biotechnol.
2020 386.

[37] Abboud M.M., Khleifat K.M., Batarseh M., Tarawneh K.A., Al-Mustafa A., Al-Madadhah M. (2007). Different optimization conditions required for enhancing
the biodegradation of linear alkylbenzosulfonate and sodium dodecyl
sulfate surfactants by novel consortium of Acinetobacter calcoaceticus
and Pantoea agglomerans, Enzyme Microb. Technol..

[38] Braga J.K., Motteran F., Silva E.L., Varesche M. B. A. (2015). Evaluation of
bacterial community from anaerobic fluidized bed reactor for the removal
of linear alkylbenzene sulfonate from laundry wastewater by 454-pyrosequence. Ecol. Eng..

[39] Macedo T.Z., Okada D.Y., Delforno T.P., Braga J.K., Silva E.L., Varesche M. B. A. (2015). The comparative advantages of ethanol
and sucrose as
co-substrates in the degradation of an anionic surfactant: microbial
community selection. Bioprocess Biosyst. Eng.
2015 3810.

[40] Zhang S., Ren Y., Zhao P., Wang X., Wang Q., Sun X. (2024). Ethanol-type
anaerobic digestion enhanced methanogenic performance by stimulating
direct interspecies electron transfer and interspecies hydrogen transfer,
Bioresour. Technol..

[41] Cruz-Salomón A., Meza-Gordillo R., Rosales-Quintero A., Ventura-Canseco C., Lagunas-Rivera S., Carrasco-Cervantes J. (2017). Biogas production from a native beverage
vinasse using a modified UASB bioreactor. Fuel.

[42] Guimarães
de Oliveira M., Marques Mourão J.M., Souza
Silva F.S., Bezerra dos Santos A., Lopes Pereira E. (2021). Effect of
microaerophilic treatment on swine wastewater (SWW) treatment: Engineering
and microbiological aspects, J. Environ. Manage..

[43] Granatto C.F., Grosseli G.M., Sakamoto I.K., Fadini P.S., Varesche M. B. A. (2021). Influence
of cosubstrate and hydraulic retention time on the removal of drugs
and hygiene products in sanitary sewage in an anaerobic Expanded Granular
Sludge Bed reactor. J. Environ. Manage..

[44] Macedo T.Z., Silva E.L., Sakamoto I.K., Zaiat M., Varesche M. B. A. (2019). Influence
of linear alkylbenzene sulfonate and ethanol on the degradation kinetics
of domestic sewage in co-digestion with commercial laundry wastewater. Bioprocess Biosyst. Eng. 2019 429.

[45] Zheng M., Han H., Shi J., Zhang Z., Ma W., Xu C. (2021). Metagenomic
analysis of aromatic ring-cleavage mechanism in nano-Fe3O4@activated
coke enhanced bio-system for coal pyrolysis wastewater treatment. J. Hazard. Mater..

[46] Atashgahi S., Hornung B., Van Der Waals M.J., Da Rocha U.N., Hugenholtz F., Nijsse B., Molenaar D., Van Spanning R., Stams A. J. M., Gerritse J., Smidt H. (2018). A benzene-degrading
nitrate-reducing microbial consortium displays aerobic and anaerobic
benzene degradation pathways. Sci. Reports 2018
81.

[47] Delforno T.P., Belgini D. R. B., Hidalgo K.J., Centurion V.B., Lacerda-Júnior G. V., Duarte I. C. S., Varesche M. B. A., Oliveira V.M. (2020). Anaerobic reactor
applied to laundry wastewater treatment:
Unveiling the microbial community by gene and genome-centric approaches. Int. Biodeterior. Biodegradation.

[48] Motteran F., de Oliveira Silva B.K., de Lima Costa J., da Silva Júnior L.P., Campos H. C. G., Tatsumi C., Magnus B.S., Leite W. R. M., Florencio L., Kato M.T. (2025). Microbial diversity, dynamics, and
metabolic analysis of microorganisms present in wastewater treatment
plants in urban areas. Brazilian J. Chem. Eng..

[49] Delforno T.P., Macedo T.Z., Midoux C., Lacerda G. V., Rué O., Mariadassou M., Loux V., Varesche M. B. A., Bouchez T., Bize A., Oliveira V.M. (2019). Comparative metatranscriptomic analysis
of anaerobic digesters treating anionic surfactant contaminated wastewater. Sci. Total Environ..

[50] Li R., Zhou M., He S., Pan T., Liu J., Zhu J. (2021). Deciphering the effect of sodium
dodecylbenzene sulfonate on up-flow
anaerobic sludge blanket treatment of synthetic sulfate-containing
wastewater. Front. Environ. Sci. Eng. 2020 155.

[51] Liu Y., Dai A., Xia L., Zhou Y., Ren T., Huang Y., Zhou Y. (2024). Deciphering
the roles of nitrogen source in sharping synchronous
metabolic pathways of linear alkylbenzene sulfonate and nitrogen in
a membrane biofilm for treating greywater. Environ.
Res..

[52] Mehta P., Deshmukh K., Dagar S.S., Dhakephalkar P.K., Lanjekar V.B. (2021). Genome sequencing and analysis of
a psychrotrophic
methanogen Methanosarcina sp. nov. MSH10X1 cultured from methane hydrate
deposits of Krishna Godavari Basin of India. Mar. Genomics.

[53] Zhou Y., Su X., Zhao W., Wang L., Fu H. (2023). Enhanced coal biomethanation
by microbial electrolysis and graphene in the anaerobic digestion. Renew. Energy.

[54] Chen Z., Song B., Guo H., Xia D., Cai Y., Wang Y., Zhao W. (2023). Metagenomic characterization of biomethane
transformation by lipid-catalyzed anaerobic fermentation of lignite. Environ. Res..

[55] Macedo T.Z., de Souza Dornelles H., do Valle Marques A.L., PalladinoDelforno T., Centurion V.B., de Oliveira V.M., Silva E.L., Varesche M. B. A. (2020). The
influence of upflow velocity and hydraulic retention time changes
on taxonomic and functional characterization in Fluidized Bed Reactor
treating commercial laundry wastewater in co-digestion with domestic
sewage. Biodegrad. 2020 311.

[56] Zheng L., Zhang C., Gao R., Zhang L., Ai W., Ulbricht M., Wei Y. (2023). Anaerobic
membrane bioreactor for
hygiene wastewater treatment in controlled ecological life support
systems: Degradation of surfactants and microbial community succession. Bioresour. Technol..

[57] Wei J., Chen B., Dong J., Wang X., Li Y., Liu Y., Guan W. (2022). Salinomycin biosynthesis reversely regulates the β-oxidation
pathway in Streptomyces albus by carrying a 3-hydroxyacyl-CoA dehydrogenase
gene in its biosynthetic gene cluster. Microb.
Biotechnol..

[58] Yadav J.S., Lawrence D.L., Nuck B.A., Federle T.W., Reddy C.A. (2001). Biotransformation
of linear alkylbenzene sulfonate (LAS) by Phanerochaete chrysosporium:
Oxidation of alkyl side-chain. Biodegradation.

[59] Eichhorn P., Knepper T.P. (2002). α, β-Unsaturated
sulfophenylcarboxylates
as degradation intermediates of linear alkylbenzenesulfonates: Evidence
for Ω-oxygenation followed by β-oxidations by liquid chromatography-mass
spectrometry. Environ. Toxicol. Chem..

[60] Wang J., Jiao D., Yuan S., Chen H., Dai J., Wang X., Guo Y., Qiu D. (2024). Comparative analysis
of microbial community under acclimation of linear alkylbenzene sulfonate
(LAS) surfactants and degradation mechanisms of functional strains. J. Hazard. Mater..

[61] Méheust R., Castelle C.J., Matheus Carnevali P.B., Farag I.F., He C., Chen L.X., Amano Y., Hug L.A., Banfield J.F. (2020). Groundwater
Elusimicrobia are metabolically diverse compared to gut microbiome
Elusimicrobia and some have a novel nitrogenase paralog. ISME J..

[62] Zhong H., Penders J., Shi Z., Ren H., Cai K., Fang C., Ding Q., Thijs C., Blaak E.E., Stehouwer C. D. A., Xu X., Yang H., Wang J., Wang J., Jonkers D. M. A. E., Masclee A. A. M., Brix S., Li J., Arts I. C. W., Kristiansen K. (2019). Impact of early events and lifestyle
on the gut microbiota and metabolic phenotypes in young school-age
children. Microbiome 2019 71.

