# Supplementary material for: Microbial Dynamics during the Removal of Linear Alkylbenzenesulfonate (LAS) under Anaerobic and Microaerobic Conditions with Different Co-substrates
Source: ACS Omega. 2026 Sep 22;11(39):58617–33. doi: 10.1021/acsomega.6c01030 (PMC13647334; doi:10.1021/acsomega.6c01030)
Supplement: Supplementary file 1 [file ao6c01030_si_001.pdf]

Supplementary material

Microbial dynamics during the removal of linear alkylbenzene sulfonate (LAS) under anaerobic and microaerobic conditions with different co-substrates

Maurício Guimarães de Oliveira[a], Vicente Elício Porfiro Sales Gonçalves da Silva[a], Amanda de Sousa e Silva [a], Fabricio Motteran[b], André Bezerra dos Santos [a,*]

[a] Department of Hydraulic and Environmental Engineering, Federal University of Ceará, Fortaleza, Ceará, Brazil

[b] Department of Civil Engineering, Centre for Technology and Geosciences, Federal University of Pernambuco, Recife, Brazil

* Corresponding author: Department of Hydraulic and Environmental Engineering. Campus do Pici, Bloco 713. Pici. CEP: 60455-900. Fortaleza – Ceará – Brazil.

E-mail: andre23@ufc.br

Figures

Figure S1 – Schematic of the continuous experimental set-up.

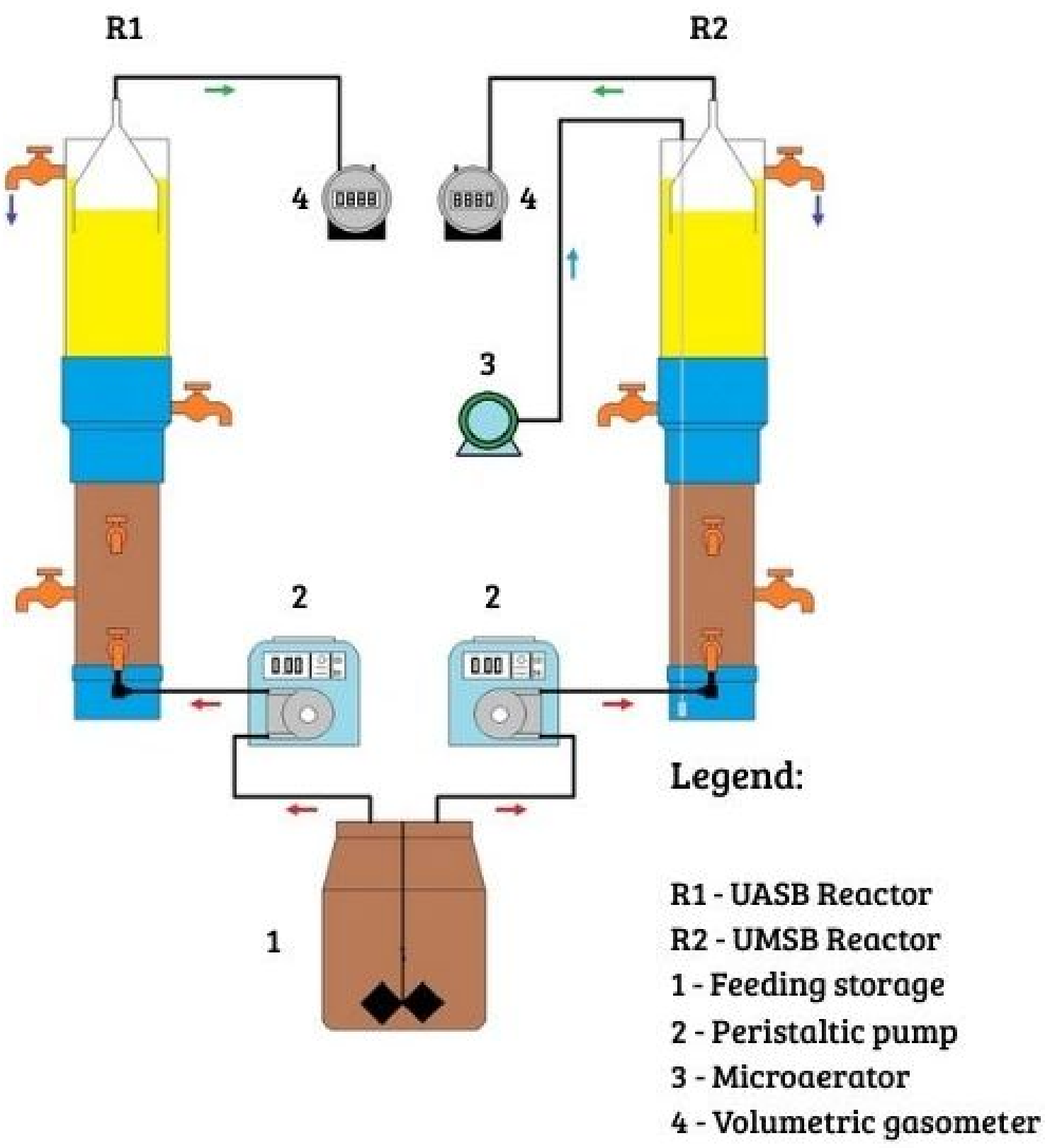

Figure S2 – Schematic of the batch experiment.

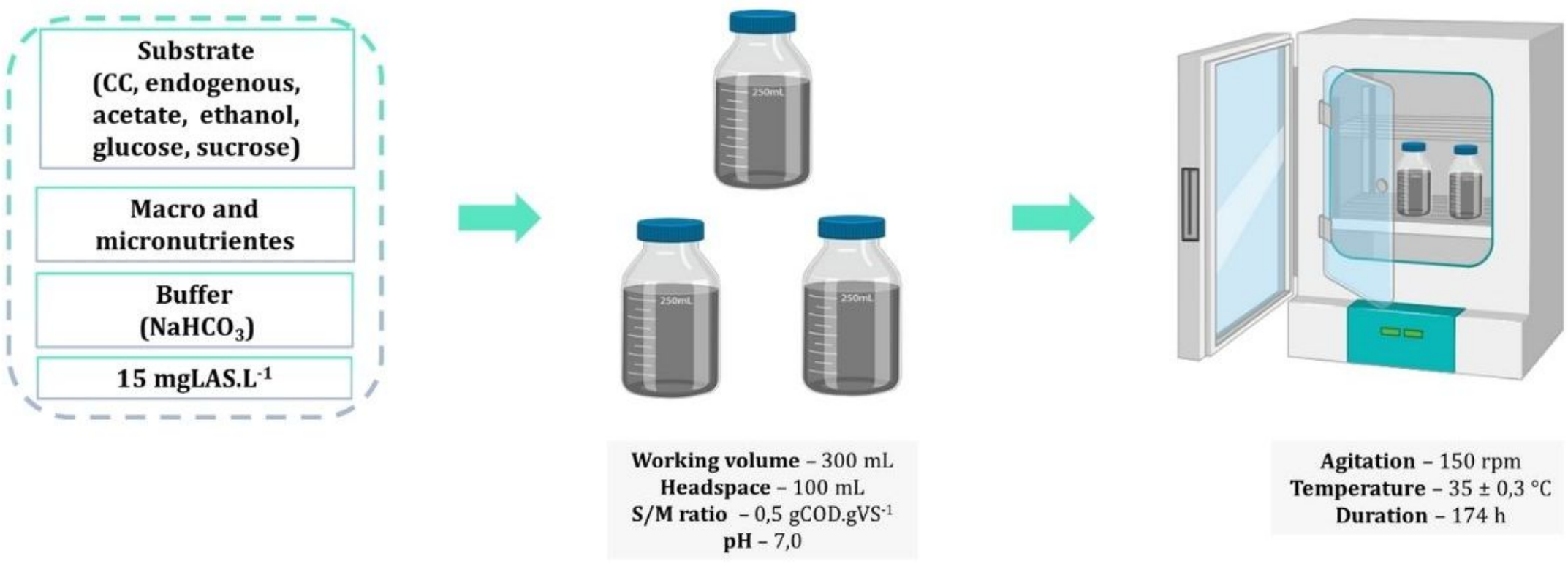

Figure S3 – LAS removal during the batch experiment.

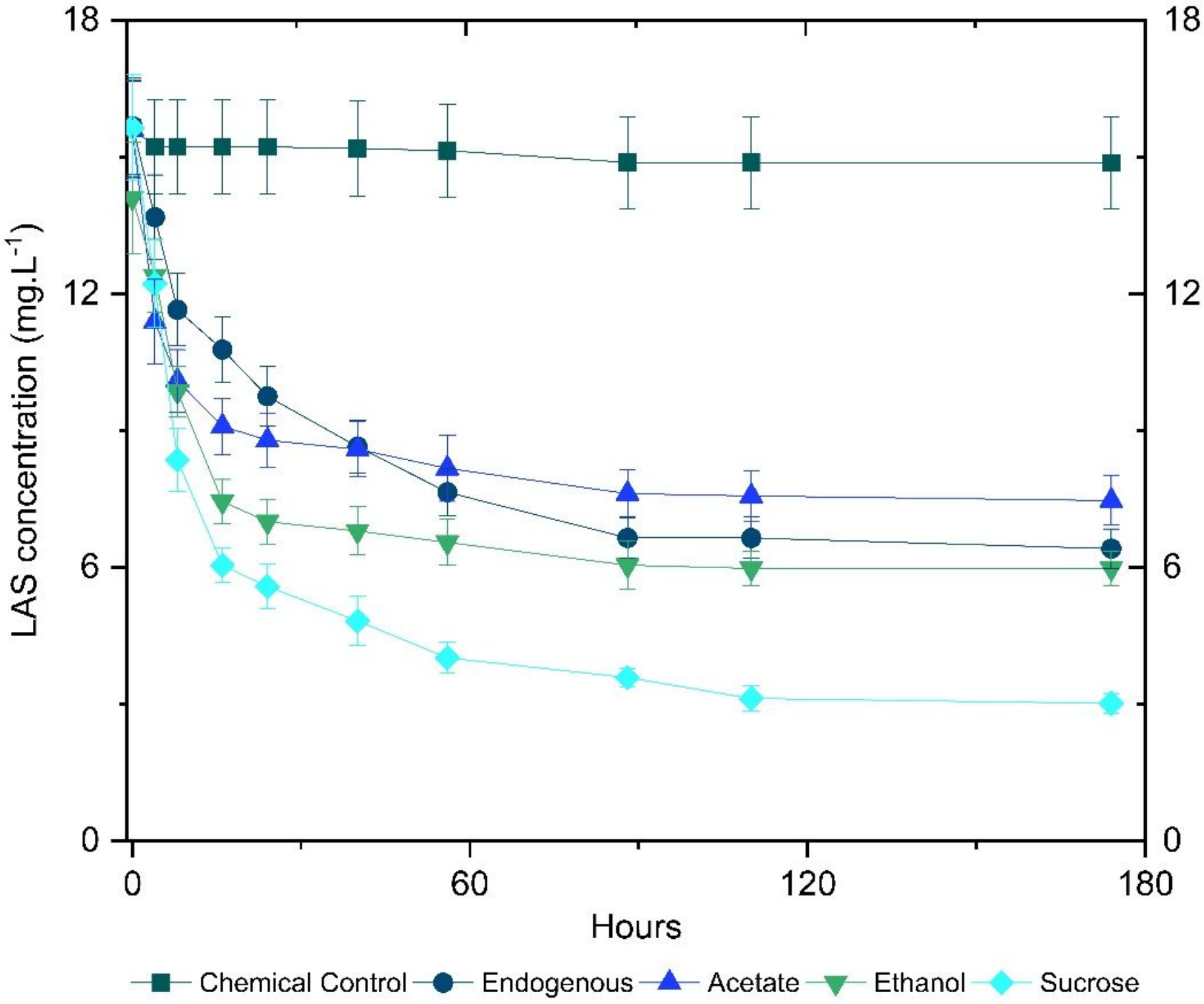

Figure S4 – Analysis of the main genes active in methanogenic pathways: a) Acetoclastic, b) Hydrogenotrophic and c) Methylotrophic.

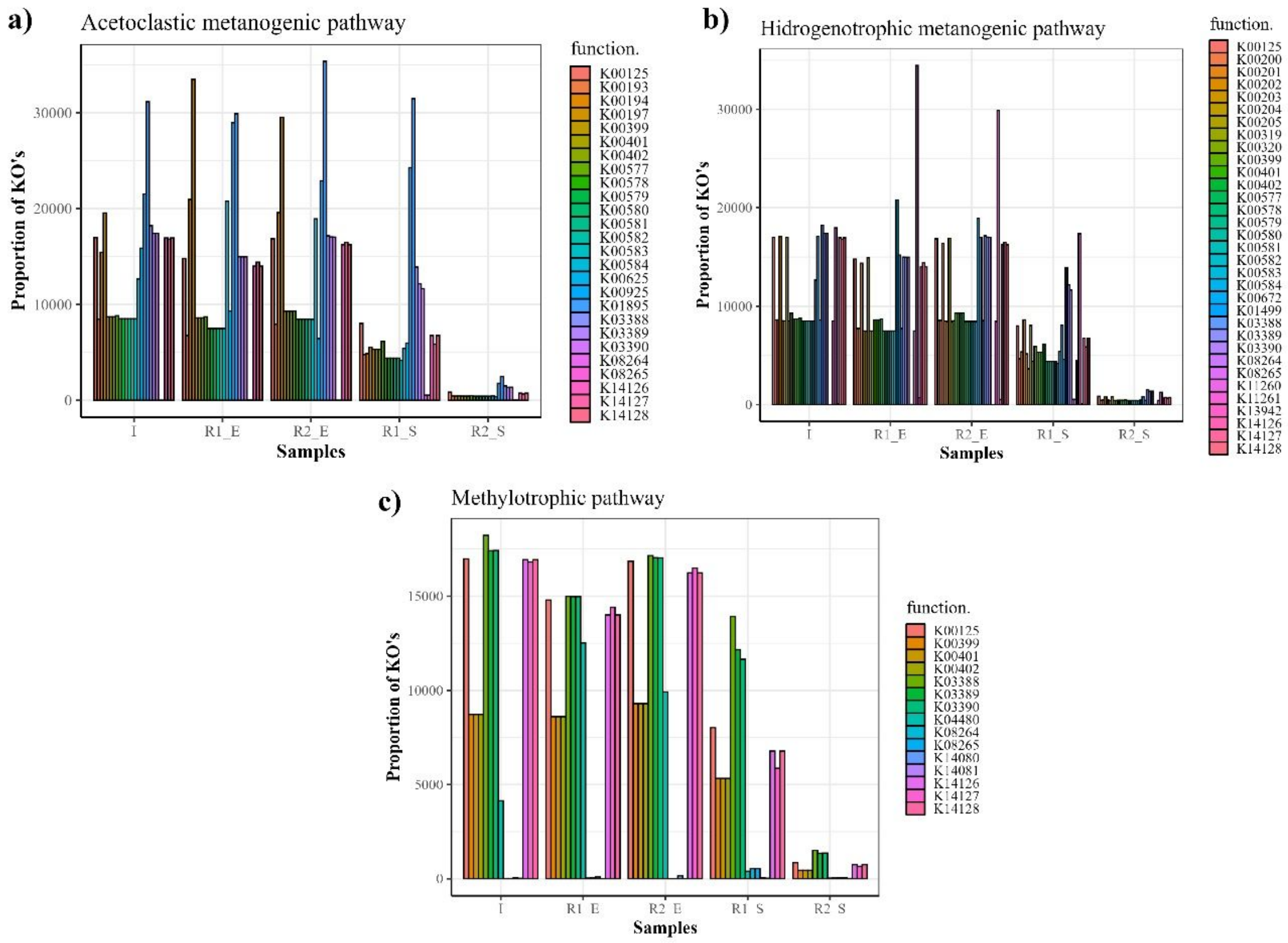

Figure S5 – Analysis of the main genes active in the a) Acetyl-CoA, b) benzene ring cleavage metabolism and c) TCA pathways.

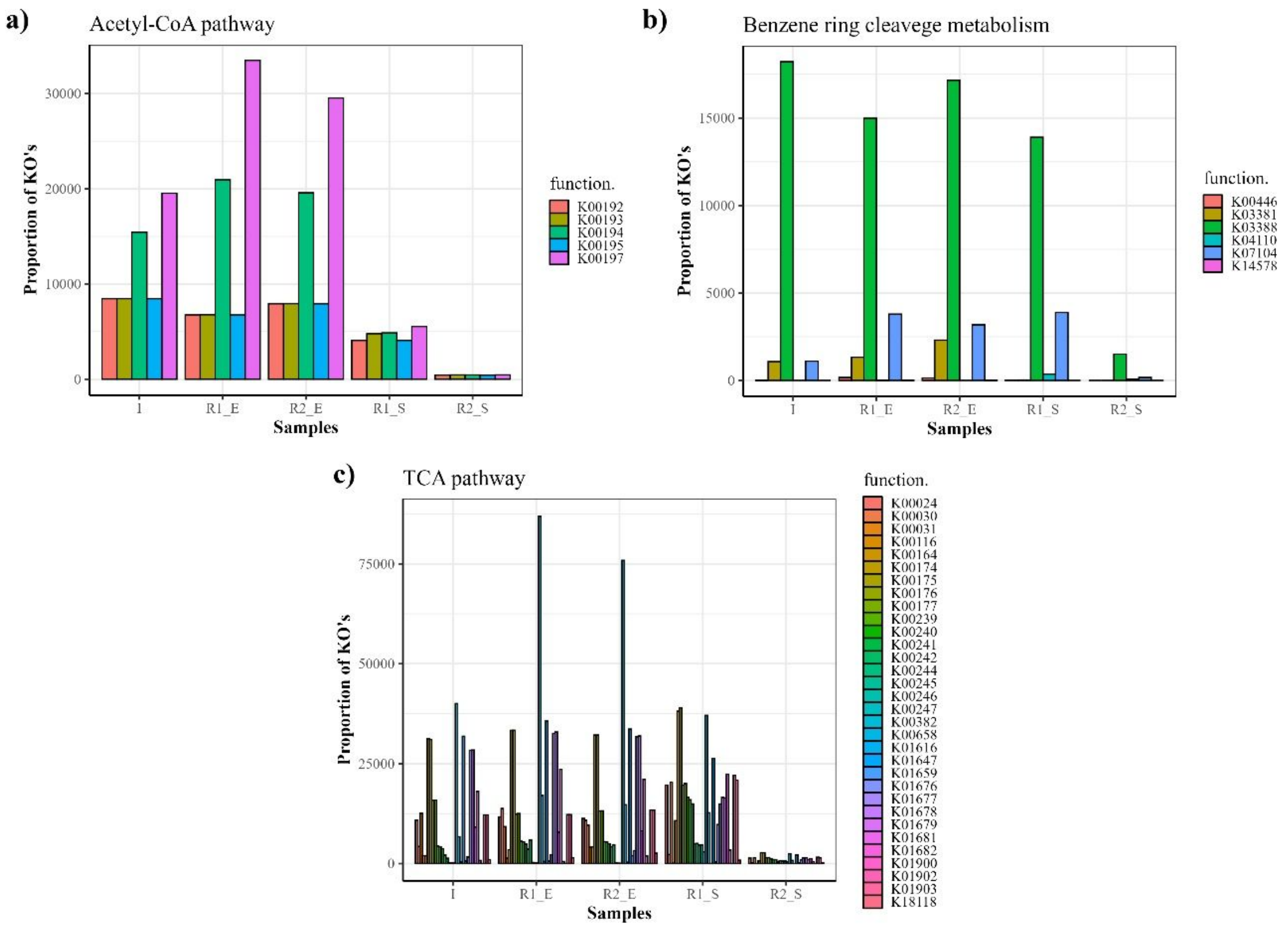

Figure S6 – Analysis of the main genes active in desulfur metabolism.

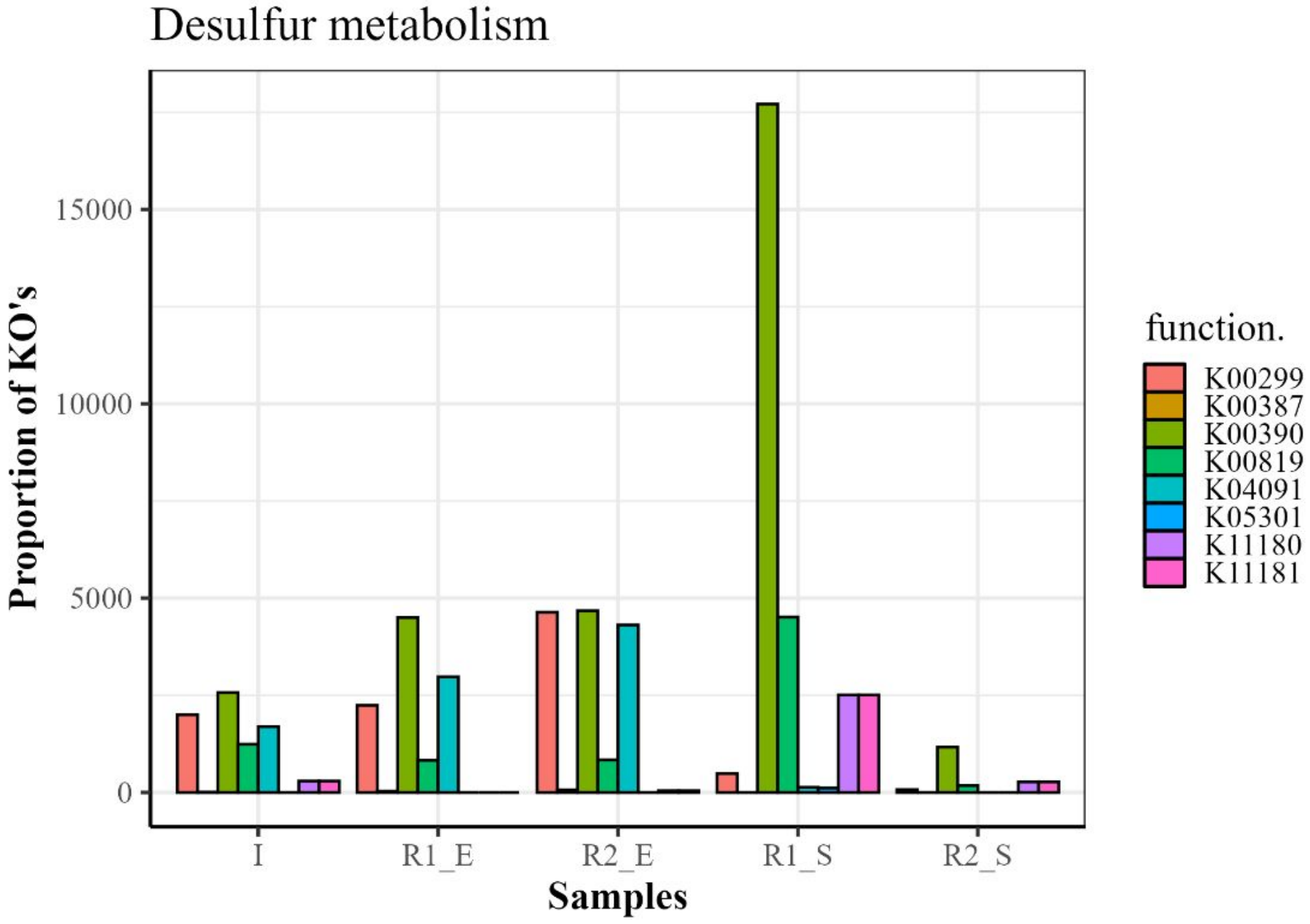

Figure S7 – Analysis of the main active genes in the a) ω-oxidation and b) β-oxidation pathways.

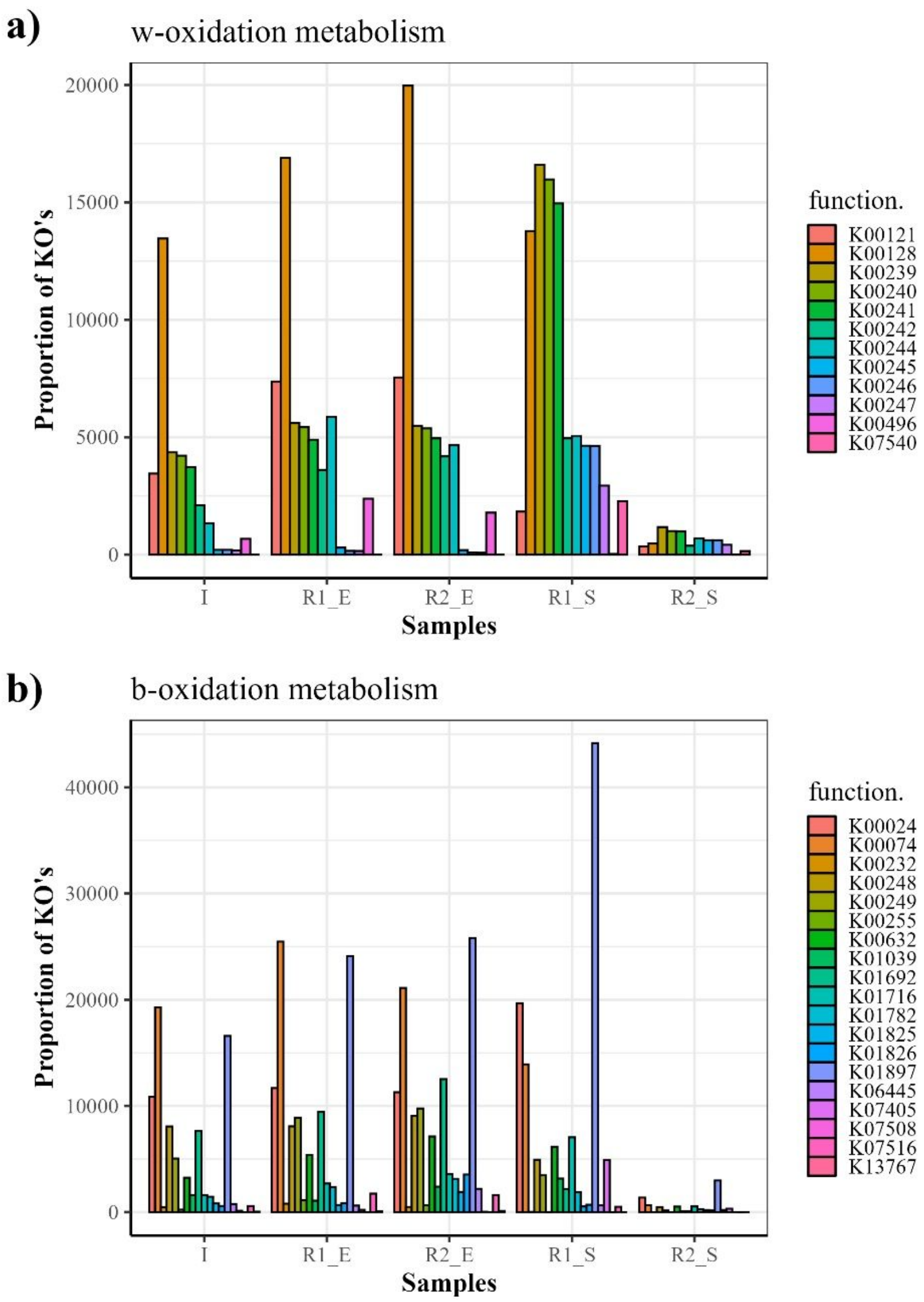

Figure S8 – Analysis of the main genes active in the a) ethanol and b) sucrose pathways.

a)

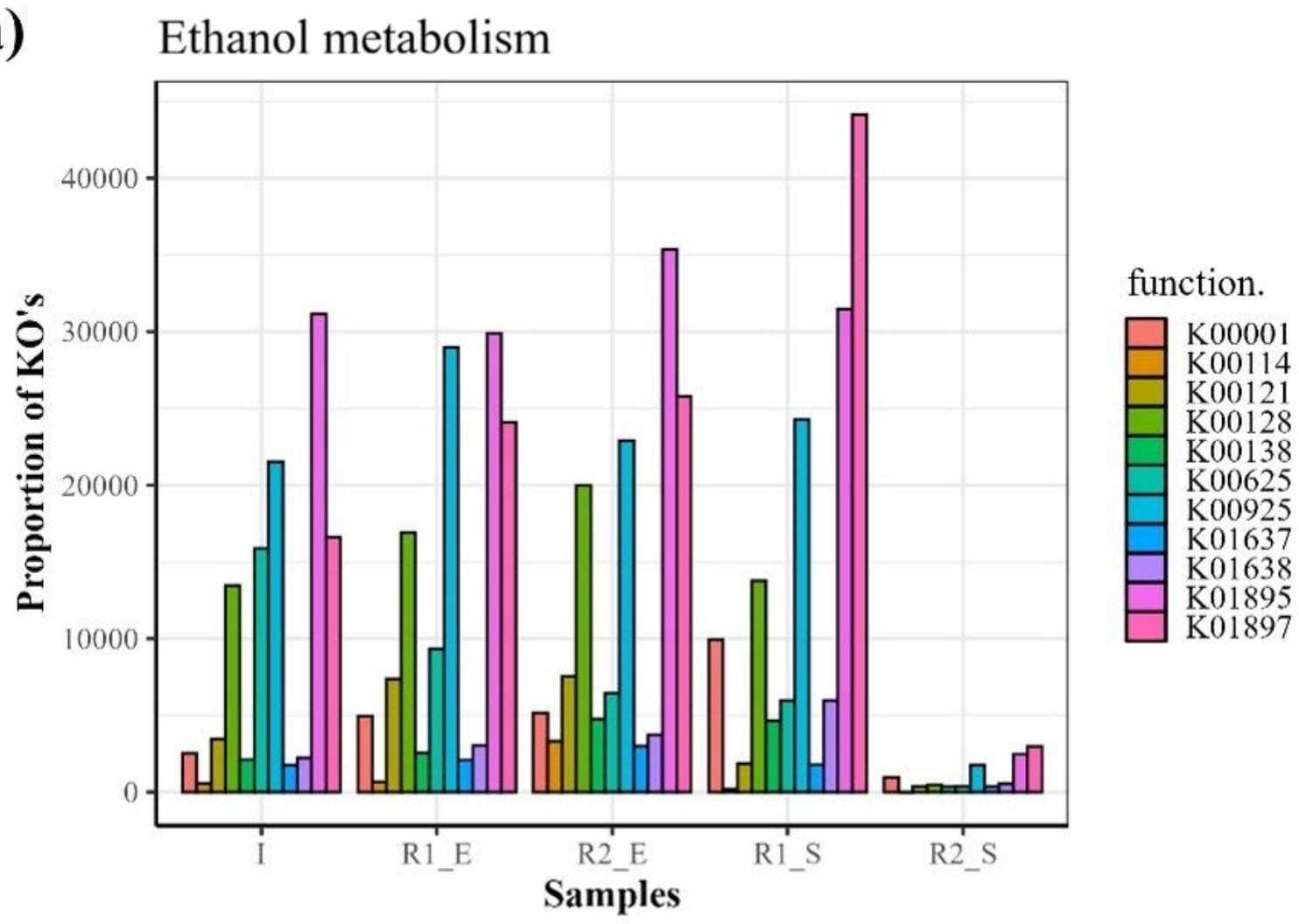

b)

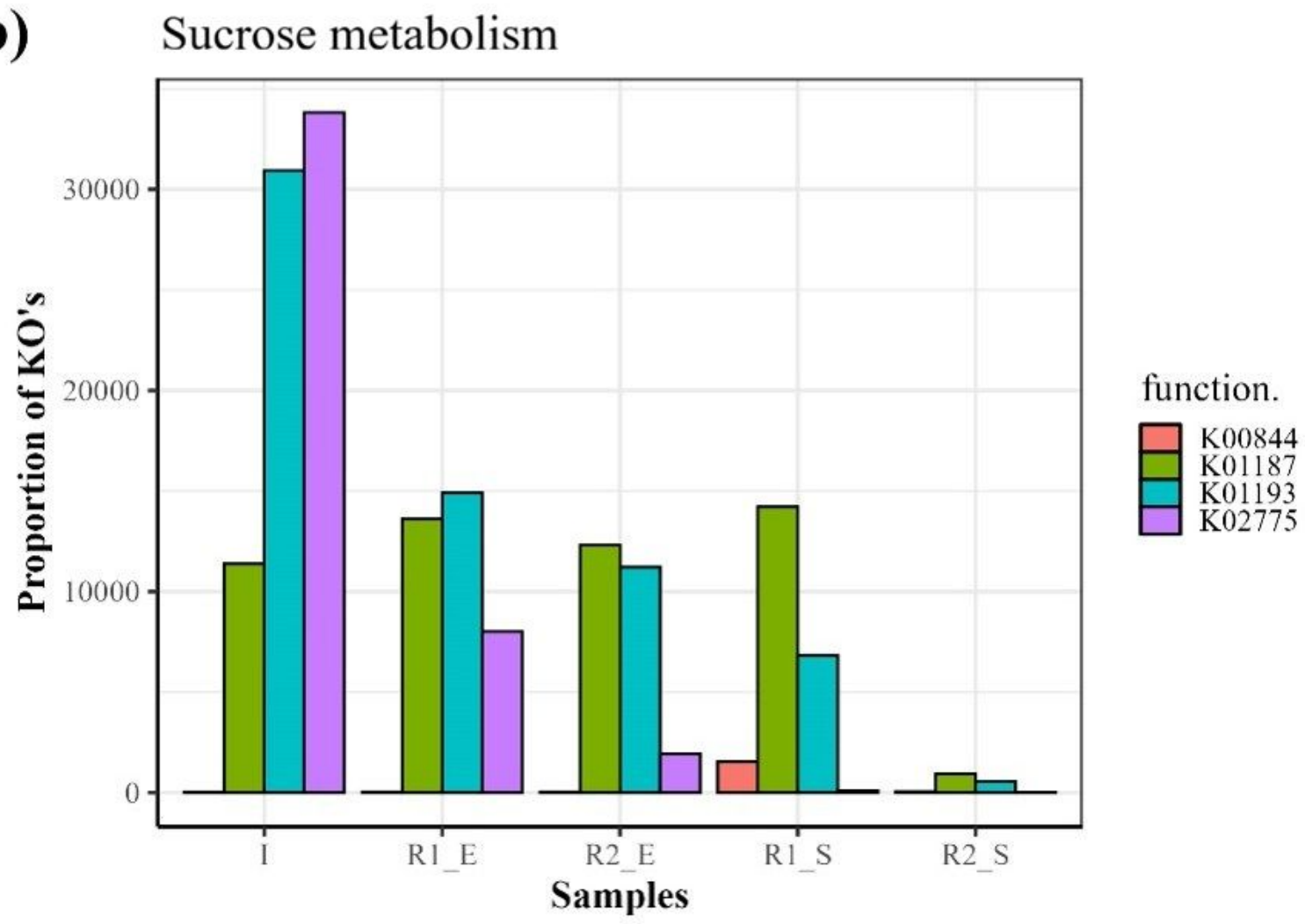

Tables

Table S1 – Comparison of the present study with previous studies discussed in the manuscript regarding LAS removal, microaeration, co-substrate selection, biogas production, and microbial community structure.

Study	Reactor or treatment system	Target compound or wastewater	Co-substrate or operational strategy	Main result reported or discussed in the manuscript	Comparison with the present study
Okada et al. [8]	UASB reactor	LAS at a concentration comparable to that used in the present study	Mixture of ethanol, methanol, and yeast extract	LAS removal of 9.0 ± 2.4% was obtained with the co-substrate mixture. Increasing co-substrate complexity increased LAS removal, reaching up to 50% with yeast extract supplementation.	In the present study, ethanol resulted in LAS removals of 19.2 ± 3.2% and 20.8 ± 4.1% in the anaerobic and microaerated reactors, respectively. Sucrose alone increased removal to 25.8% in the anaerobic reactor and 34.3% in the microaerated reactor under an HRT of 8 h.
Costa et al. [9]	Anaerobic system treating LAS-containing wastewater	LAS	Anaerobic treatment with nitrate, sulfate, and iron(III) as electron acceptors	*Bacillota* was predominant in the microbial community. *Pseudomonadota* was also reported as an abundant phylum in anaerobic systems treating surfactant-containing wastewater, particularly with sucrose as a co-substrate.	*Bacillota* predominated in the ethanol-fed reactors of the present study. After sucrose addition, *Pseudomonadota* became dominant, particularly in the microaerated reactor, coinciding with increased LAS removal.
Nascimento et al. [17]	High-rate anaerobic system under microaerated conditions	Parabens	Microaeration	An airflow of 4 mL·min⁻¹ increased contaminant removal to 90–94%, compared with less than 30% under strictly anaerobic conditions. Microaeration improved treatment performance but compromised methane purity.	The present study also showed that microaeration could improve contaminant removal while decreasing methane concentration in the biogas. However, the improvement in LAS removal depended strongly on the co-substrate and was significant only when sucrose was supplied.
Macedo et al. [39]	Anaerobic fluidized-bed reactor	LAS	Ethanol or a sucrose–ethanol mixture	Higher LAS removal was obtained with ethanol than with the sucrose–ethanol mixture.	The opposite tendency was observed in the present study, in which sucrose promoted higher LAS removal than ethanol. This

					difference may be associated with reactor configuration and operating conditions.
Oliveira et al. [42]	Microaerophilic treatment system	Swine wastewater	Microaerophilic treatment	Microaeration enhanced organic matter removal but compromised methane purity.	A similar behavior was observed in the present study. Methane concentrations were lower in the microaerated reactor, averaging 36.6% with ethanol and 32.7% with sucrose, although volumetric methane production was maintained or increased.
Delforno et al. [47]	Expanded granular sludge bed reactor	Effluent containing 14 $mg \cdot L^{-1}$ LAS	Ethanol and methanol	A high abundance of *Bacillota* was observed in the reactor biomass.	*Bacillota* also predominated in the ethanol-fed reactors of the present study, mainly through the classes *Clostridia* and *Bacilli* and the families *Eubacteriaceae* and *Carnobacteriaceae*.
Present study	Conventional UASB reactor and microaerated UASB reactor, designated UMSB	Synthetic domestic wastewater containing 15 $mg \cdot L^{-1}$ LAS	Ethanol or sucrose at 1 $gCOD \cdot L^{-1}$; microaeration dose of 0.1 L $O_2 \cdot Lfeed^{-1} \cdot d^{-1}$; HRT of 8 h	With ethanol, LAS removal was 19.2 ± 3.2% in the UASB reactor and 20.8 ± 4.1% in the UMSB reactor, with no significant difference. With sucrose, removal increased to 25.8% and 34.3%, respectively. Microaeration with sucrose promoted a less diverse but more specialized community associated with *Pleomorphomonas*, *Desulfovibrio*, oxidative pathways, sulfur metabolism, and maintenance of methanogenic functions.	The combined use of sucrose and microaeration provided the highest LAS removal. Unlike the previous studies discussed in the manuscript, the present work jointly evaluated co-substrate selection, controlled microaeration, reactor performance, methane production, microbial community structure, and predicted metabolic pathways related to LAS transformation.

Abbreviations: COD, chemical oxygen demand; HRT, hydraulic retention time; LAS, linear alkylbenzene sulfonate; UASB, upflow anaerobic sludge blanket; UMSB, upflow microaerobic sludge blanket.
